# M2 macrophage-derived exosomes delivering haptoglobin and interleukin-10 plasmids for synergistic therapy of intracerebral hemorrhage

**DOI:** 10.1016/j.bioactmat.2026.01.047

**Published:** 2026-02-03

**Authors:** Ke Wang, Jin Li, Meng He, Liping Wang, Xing Guo, Shaobing Zhou

**Affiliations:** a Institute of Biomedical Engineering, College of Medicine, Southwest Jiaotong University, Chengdu, 610031, China; b Key Laboratory of Advanced Technologies of Materials Ministry of Education, School of Materials Science and Engineering, Southwest Jiaotong University, Chengdu, 610031, China

**Keywords:** Drug delivery, Nanocarriers, M2 macrophage-derived exosomes, Hematoma clearance, Neuroinflammation alleviation

## Abstract

Intracerebral hemorrhage (ICH) carries high mortality and disability rates, driven not only by the initial bleeding but also by secondary neurotoxicity from blood derived components such as hemoglobin. An effective strategy during the subacute phase must therefore address both hematoma clearance and neuroinflammation. To achieve this dual action therapy, we engineered a novel nanoplatform (M2-exo@HI) by encapsulating a plasmid co-expressing haptoglobin (Hp) and interleukin-10 (IL-10) within M2 macrophage-derived exosomes. Leveraging the innate inflammatory homing of macrophage exosomes, M2 exo@HI delivers the HI plasmid to the ICH site, where it is primarily internalized by M1 microglia. The expressed Hp binds hemoglobin, reducing neurotoxicity and exert neuroprotective effects. Simultaneously, IL-10 polarizes M1 microglia to the neuroprotective M2 phenotype, thereby removing hematoma and alleviating neuroinflammation via anti-inflammatory cytokine production. Consequently, M2-exo@HI significantly reduced hematoma volume, improved long-term neurological function, and enhanced blood-brain barrier (BBB) repair. Transcriptome analysis further elucidated the genetic mechanisms underlying this synergistic effect. This work presents a promising co-delivery strategy for enhancing hemorrhagic stroke therapy.

## Highlights

• M2 macrophage-derived exosomes to deliver an Hp and IL-10 encoding plasmid for intracerebral hemorrhage therapy.
• The nanoplatform uses inflammatory homing to target the hemorrhage site, delivering its plasmid primarily to M1 microglia.
• Expressed haptoglobin clears toxic hemoglobin, while IL-10 shifts M1 microglia to a protective M2 phenotype.
• This synergistic strategy reduced hematoma volume, enhanced BBB repair, and improved long-term neurological function.

## Graphical abstract

**Figure:**
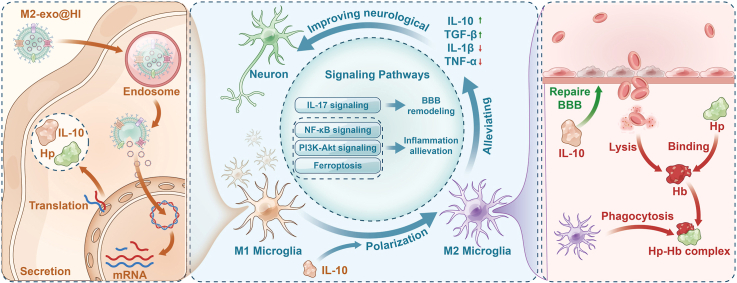

## 1 Introduction

Intracerebral hemorrhage (ICH) ranks among the most disabling and fatal cerebrovascular diseases worldwide, accounting for 10–20 % of all strokes yet bearing a disproportionately high mortality rate of approximately 40 % [1]. Although surgical hematoma evacuation remains a therapeutic cornerstone, its efficacy is often limited by heterogeneous hemorrhage patterns, deep or inaccessible clot locations, and the risk of rebleeding, contributing to inconsistent functional outcomes across clinical trials [2]. Current hyperacute pharmacological management of ICH focuses on hemodynamic stabilization, complication prevention, and intracranial pressure control. However, the absence of disease-modifying agents that directly mitigate hematoma expansion significantly undermines the overall effectiveness of drug therapy [[3], [4], [5]]. These challenges are compounded by the complex pathophysiology of ICH, particularly the progressive secondary brain injury that occurs after the initial bleed. Consequently, the urgent need for effective ICH therapeutics persists for subacute management to limit secondary injury and improve outcomes.

The pathophysiology of secondary brain injury following ICH critically involves the lysis of red blood cells (RBCs) within the hematoma, releasing hemoglobin (Hb). Degradation of Hb liberates the potent cytotoxins heme and iron. Cell-free Hb itself acts as a significant neurotoxin, driving oxidative stress, inflammation, blood-brain barrier (BBB) disruption, and neuronal death [6]. Haptoglobin (Hp), an endogenous acute-phase protein produced primarily in the liver and spleen, offers a natural defense mechanism by binding free Hb with high affinity [7]. The resulting Hb-Hp complex sequesters heme iron, mitigating its pro-oxidant and cytotoxic effects. However, the therapeutic potential of Hp is severely limited by its negligible presence within the central nervous system (CNS) and minimal penetration across the BBB [8].

Compounding the damage from Hb toxicity, dysregulated neuroinflammation plays a pivotal role in ICH pathogenesis. Microglia, the resident immune cells of brain, rapidly activate and polarize into distinct phenotypes post-ICH: the pro-inflammatory and neurotoxic M1 state or the reparative and neuroprotective M2 state [9]. M2 microglia promotes tissue repair by secreting anti-inflammatory cytokines. More importantly, M2 microglia can remove hematoma by CD36-mediated erythrocyte phagocytosis and CD163-mediated Hb-Hp complex elimination [10]. Conversely, excessive M1 activation propagates secondary damage through pro-inflammatory cytokine release and oxidative stress. As a key regulator, interleukin-10 (IL-10) can promote the beneficial M2 phenotype to enhance phagocytic capacity of microglia and protect neurons [11,12]. Given the multifaceted nature of ICH injury, a combined therapeutic strategy leveraging the synergistic actions of IL-10 and Hp offers significant potential for enhanced neuroprotection and improved functional outcomes in hemorrhagic stroke.

Recent advances highlight macrophage-derived exosomes as promising natural nanocarriers for CNS drug delivery. Inheriting parental membrane properties and cytoplasmic contents, these vesicles exhibit excellent biocompatibility, low immunogenicity, and inflammation-targeting capability [13,14]. Besides, exosomes efficiently transport diverse therapeutic cargoes, including genetic material, between cells [15,16]. Critically, exosomes derived from M2-polarized macrophages (M2-exo) display surface proteins (e.g., mannose receptors) that facilitate their selective uptake by M1 microglia via ligand-receptor interactions [17,18].

**Fig. 1:**
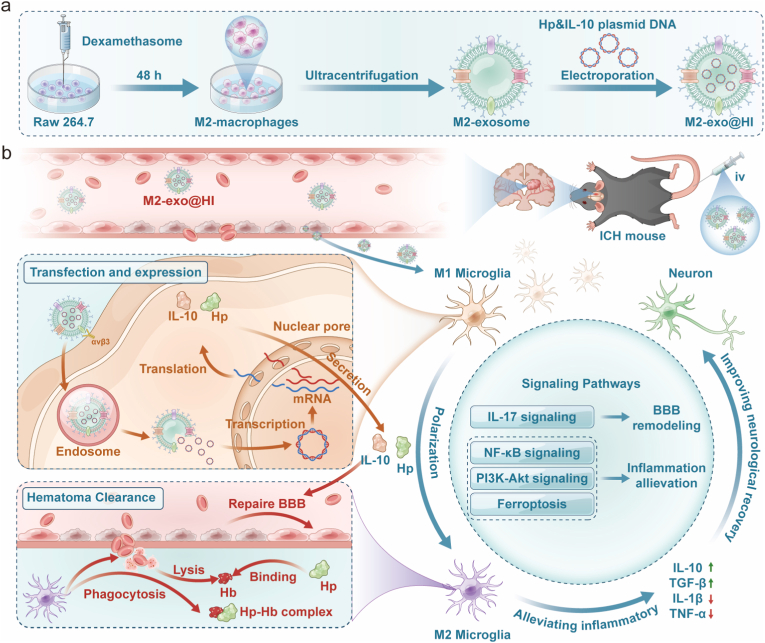
Schematic illustration of the preparation of M2-exo@HI and its mediated therapeutic mechanisms and signaling pathways. (a) RAW264.7 macrophages were polarized to M2 phenotype using DEX, followed by M2-exo isolation from cell supernatant via differential centrifugation. M2-exo@HI was prepared through encapsulating HI into M2-exo by electroporation. (b) M2-exo@HI crossed the BBB and localized to microglia in the hemorrhagic brain, delivering the HI plasmid into the nucleus. This prompted expression and secretion of Hp and IL-10 by microglia. The released Hp inhibited Hb toxicity by binding to Hb. IL-10 shifted microglial polarization from the pro-inflammatory M1 phenotype toward the reparative M2 phenotype, removing hematoma by engulfing erythrocyte and Hb-Hp complex, and decreasing pro-inflammatory cytokine levels—collectively enhancing neuroprotection and BBB repair. These beneficial outcomes were linked to the inhibition of the IL-17 and NF-κB signaling pathways and activation of the PI3K-Akt and ferroptosis pathways.

Building upon these insights and our extant research methodologies [19], we propose an innovative synergistic therapeutic platform for ICH: M2 macrophage-derived exosomes loaded with plasmids encoding Hp and IL-10 (M2-exo@HI) (Fig. 1). This engineered nanoplatform leverages the natural tropism of M2-exo to cross the BBB and accumulate at the hemorrhagic site. Upon internalization by M1 microglia, localized expression of IL-10 drives their polarization towards the protective M2 phenotype, enhancing hematoma clearance, neuroprotection, and BBB restoration. Concurrently, the expressed Hp neutralizes cytotoxic Hb, synergistically reducing inflammation and neurotoxicity. By simultaneously addressing the dual pathological pathways of Hb-mediated toxicity and maladaptive neuroinflammation, the M2-exo@HI system presents a novel and highly promising strategy for improving outcomes in hemorrhagic stroke.

## 2 Materials and methods

### 2.1 Cell lines and culture conditions

Murine macrophage RAW 264.7 cells, microglial BV2 cells, endothelial cells **(**ECs), 293T cells, and cerebral vascular endothelial cells (bEnd.3) were maintained in Dulbecco's modified Eagle's medium (DMEM, Hyclone) supplemented with 10 % fetal bovine serum (FBS, Biological Industries). Rat adrenal pheochromocytoma PC12 cells were cultured in RPMI-1640 medium (Hyclone). All cell lines were incubated at 37 °C under 5 % CO_2_ humidified atmosphere.

### 2.2 Macrophage polarization

To polarize RAW 264.7 cells to M2 phenotype, 2 μM dexamethasone (DEX) (Solarbio, China) was added to the culture medium without FBS for 48 h. To test the polarization efficiency, macrophages were labeled with CD206-PE (1:100, eBioscience) and CD86-PE (1:100, Abcam) for flow cytometry analysis. The supernatant was collected and measured with mouse IL-10, IL-4, IL-1β, and TNF-α enzyme-linked immunosorbent assay (ELISA) kits (Shanghai Enzyme-linked Biotechnology Co., Ltd., China), respectively.

### 2.3 Collection of exosomes

The culture medium was centrifuged at 1500×*g* for 10 min and 3500×*g* for 30 min to remove dead cells, apoptotic bodies and cell fragments. The supernatant was concentrated by ultrafiltration (10000×*g*, 1 h) to remove micro-vesicles, and filtered using a 0.22-μm filter (Millipore, USA) to further remove cells and debris. Subsequently, the exosomes were collected via ultracentrifugation at 150,000×*g* for 2 h (Beckman Coulter Optima XPN ultracentrifuge, sw70Ti). All the process was carried out at 4 °C. M2-exo were resuspended and stored in PBS at −80 °C prior to use.

### 2.4 Characterization of exosomes

Western blot was performed to detect the protein expressions in the membranes and exosomes derived from RAW 264.7 cells and M2 macrophages. The protein content was quantified by bicinchoninic acid (BCA) protein assay (Beyotime), and an equal amount of protein (20 μg) was added to the SDS-PAGE. Then the gel was transferred to the polyvinylidene difluoride (PVDF) membrane and incubated with primary antibodies including CD9 (1:1000, Beyotime), Tsg101 (1:1000, Beyotime), Calnexin (1:2500, Beyotime), and β-actin (1:10000, Abcam) at 4 °C overnight. Afterward, the membrane was incubated with horseradish peroxidase (HRP)-conjugated secondary antibodies at room temperature for 1 h, followed by reacting with the enhanced chemiluminescence substrate. The protein bands were imaged by a chemiluminescence imager (Monna, QuickChemi 5100).

### 2.5 Preparation of M2-exo@HI

M2-exo (100 μL, 1 mg/mL), HI plasmid (circular pBudCE4.1, 100 μg, TsingkeBiotechnology., Ltd.), and 100 μL PBS were added to the electroporation cuvettes (Bio-Rad 165-2088). And electroporation was performed under the condition of 250 V and 350 μF on an electroporation system (Bio-Rad). The solution was then incubated at 37 °C for 30 min to allow the recovery of the exosome membrane. The excess HI plasmid was removed by centrifugation. The M2-exo@HI was resuspended and stored in PBS at 4 °C for further use.

### 2.6 Characterization of M2-exo@HI

The particle number was determined by nanoparticle tracking analysis (NTA, ZetaView, Particle Metrix). The particle size and zeta potential were measured by dynamic light scattering (DLS, Malvern Nano-ZS90, UK). The morphology was examined by transmission electron microscope (TEM, JSM-7800F, Japan). The concentration of HI plasmid in M2-exo was determined by the microspectrophotometer (NanoDrop, Thermo Fisher Scientific). The HI plasmid loading efficiency (LE) was calculated as Equation (1).

(1) $$\text{LE}\;\left(\%\right)=\frac{\text{HI}\;\text{plasmid}\;\text{in}\;exo\;\left(\text{ng}/\mu L\right)}{\text{Total}\;\text{amount}\;\text{of}\;\text{DNA}\;\text{invested}\left(\text{ng}/\mu L\right)}\times 100\%$$

The integrity of plasmids post-electroporation was assessed via DNA agarose gel electrophoresis. The procedure was performed as follows: HI plasmids were initially co-electroporated with M2-exo at voltages of 150, 250, 350, 450, 550, or 650 V. After a 30-min incubation at 37 °C, M2-exo@HI complexes were collected by centrifugation. Each sample was lysed with 20 μL of RIPA buffer at 4 °C for 30 min, followed by plasmid DNA recovery through centrifugation. The extracted DNA was then analyzed by agarose gel electrophoresis, and the gel images were recorded. Plasmid expression activity was evaluated by transfecting BV2 cells and subsequently examining fluorescence under microscopy. M2-exo@HI complexes prepared at different electroporation voltages were used for transfection. Fluorescence images were captured 24 h after transfection.

### 2.7 Validation of HI plasmid construction and evaluation of M2-exo delivery efficiency

To confirm the successful construction of the HI plasmid and evaluate the delivery capability of M2-exo, the expression of the plasmid-encoded fluorescent protein was observed in 293T cells using fluorescence microscopy 24 h after transfection. The procedure was conducted as follows: 293T cells were first seeded in a 24-well plate at a density of 5 × 10^4^ cells per well and cultured for 24 h. Then, the medium was replaced with 1 mL of Opti-MEM™ reduced serum medium (Gibco), followed by the addition of Lipo 2000@Vector, Lipo 2000@HI, and M2-exo@HI. The amount of DNA added per well was 5 μg. After 4 h of incubation, the medium was replaced with fresh complete medium to minimize cytotoxicity. Finally, transfection efficiency was evaluated using fluorescence microscopy 24 h post-transfection.

### 2.8 In vitro drug release

M2-exo@HI (100 μg/mL) were loaded into a dialysis bag, which was then placed in centrifuge tubes containing buffer solutions at pH 6.8 and pH 7.4, respectively. The tubes were incubated at 37 °C with shaking at 100 rpm. Samples (10 μL) were collected at predetermined time points (0.25, 0.5, 1, 2, 4, 8, 12, and 24 h) and analyzed using a microspectrophotometer.

### 2.9 Cytocompatibility assay

The ECs and BV2 cells were seeded into a 48-well plate (4 × 10^4^ cells per well) and treated with M2-exo@HI at concentrations of 25 μg/mL, 50 μg/mL, 100 μg/mL, 200 μg/mL, and 400 μg/mL, respectively. After 24 h, cells were stained with Calcein-AM (2 mM) and PI (4 mM) for 30 min of incubation in the dark, and observed under a fluorescence microscope (Zeiss Axio Observer).

### 2.10 Cell viability

RAW264.7 macrophages were plated in a 48-well plate (4 × 10^4^ cells per well) and incubated with DEX at concentrations of 200 nM, 500 nM, 1 μM, and 2 μM for 24 h. Then the medium was removed and added with 200 μL of CCK8 solution (10 % CCK8, 90 % medium, v/v) for 30 min of incubation. The absorbance of the supernatant was measured at 450 nm using a microplate reader (Thermo Fisher Scientific, LUX, USA). Cell viability was calculated by Equation (2).

(2) $$\text{Cell}\;\text{viability}\;\left(\%\right)=\frac{\text{OD}\;\left(\text{sample}\right)-\text{OD}\;\left(\text{blank}\right)}{\text{OD}\;\left(\text{control}\right)-\text{OD}\;\left(\text{blank}\right)}\times 100\%$$

### 2.11 Hemocompatibility evaluation

Blood compatibility of M2-exo@HI was determined by the hemolysis test and coagulation test. For hemolysis test, whole mouse blood was prepared into 2 % red blood cell suspension and treated with M2-exo@HI. 0.1 % Triton X-100 solution and PBS were used as positive control and negative control, respectively. After 1 h of incubation at 37 °C, blood was centrifuged at 1500 rpm for 10 min. The absorption of upper serum was measured at 540 nm using microplate reader and the hemolysis rate was calculated by Equation (3).

(3) $$\text{Hemolysis}\;\text{rate}\;\left(\%\right)=\frac{\text{OD}\;\left(\text{sample}\right)-\text{OD}\;\left(\text{negative}\;\text{control}\right)}{\text{OD}\;\left(\text{positive}\;\text{control}\right)-\text{OD}\;\left(\text{negative}\;\text{control}\right)}\times 100\%$$

For whole blood clotting assay, mouse blood was collected and quickly mixed with M2-exo@HI. Deionized water and PBS were used as the positive control and negative control, respectively. After incubation at 37 °C for 90 s, 10 mL of deionized water was gently added to the sample. The mixture was centrifuged at 1000 rpm for 1 min. The absorption of the upper serum was measured and the blood coagulation index (BCI) was calculated by Equation (4).

(4) $$\text{BCI}\;\left(\%\right)=\frac{\text{OD}\;\left(\text{sample}\right)}{\text{OD}\;\left(\text{positive}\;\text{control}\right)}\times 100\%$$

### 2.12 Cellular uptake of M2-exo@HI

Confocal laser scanning microscope (CLSM) and flow cytometry were used to analyze the phagocytosis of M1-type microglia on M2-exo. Firstly, BV2 cells were treated with 200 ng/mL of lipopolysaccharide (LPS) for 24 h to simulate the ICH microenvironment. The polarization of microglia was subsequently validated using flow cytometry, with CD86 and CD206 serving as markers for M1 and M2 phenotypes, respectively. Then exosomes were loaded with either indocyanine green (ICG) or Rhodamine B (RhB) (each at 1 mg/mL) via electroporation, followed by a 30-min of incubation to allow membrane resealing. The samples were subsequently centrifuged to collect M2-exo@ICG and M2-exo@RhB, respectively. For CLSM observation, 100 μL ICG and M2-exo@ICG were incubated with M1 microglia for 0.25 h, 0.5 h, 1 h and 2 h. Then cells were washed, fixed, stained with DAPI and observed by CLSM (A1R+, Nikon, Japan). For flow cytometry analysis, 100 μL of RhB and M2-exo@RhB were incubated with M1 microglia for 0.25 h, 0.5 h, 1 h and 2 h cells after phagocytosis were centrifuged to remove the extracellular RhB or M2-exo@RhB, and resuspended with PBS. The RhB fluorescence signal was detected by flow cytometry.

The phagocytosis and release kinetics of M2-exo@RhB were further investigated. Briefly, BV2 cells were treated with 4 % red blood cell lysate for 6 h to simulate hemorrhagic stimulation. Subsequently, M2-exo@RhB was added to the culture medium at a concentration of 1 mg/mL. At predetermined time points (0.25, 0.5, 1, and 2 h), the culture supernatant and cells were collected separately. The supernatant was centrifuged using ultrafiltration tubes to isolate the released RhB, while the cells were lysed with RIPA buffer to extract the phagocytosed RhB. The fluorescence intensity of RhB in both fractions was measured with a microplate reader, allowing evaluation of the phagocytosis and release kinetics of M2-exo@RhB in BV2 cells under ICH-mimicking condition.

### 2.13 Cell transfection and protein expression

To compare the transfection efficiency between the conventional reagent Lipo 2000 and M2-exo in M1 microglia, BV2 cells were first polarized with LPS (200 ng/mL) for 24 h to induce the M1 phenotype. Subsequently, the cells were transfected with either Lipo2000@HI or M2-exo@HI at DNA doses of 1 μg, 2.5 μg, 5 μg, 10 μg, and 15 μg. After 24 h of transfection, the concentrations of Hp and IL-10 in the cell culture supernatants were quantified using ELISA kits (Elabscience). The temporal expression profile of Hp and IL-10 mediated by M2-exo@HI was further examined. M1-polarized microglia were transfected with 5 μg of M2-exo@HI and incubated for 12, 24, 48, and 72 h. Protein expression was then evaluated using fluorescence microscopy, Western blot, quantitative polymerase chain reaction (qPCR), and ELISA, respectively.

### 2.14 Western blotting

For all samples, the total cells were lysed by RIPA buffer and measured by BCA protein analysis kit. An equal amount of protein (20 μg) was added to SDS-PAGE. Then the gel was transferred to PVDF membrane and incubated with primary antibodies including rabbit anti-HP (1:1000, ABclonal), rabbit anti-IL-10 (1:1000, ABclonal), rabbit anti-CXCR4 (1:1500, ABclonal), rabbit anti-CD163 (1:1500, ABclonal), rabbit anti-IGF2 (1:750, ABclonal), rabbit anti-IKKε (1:1000, ABclonal) and rabbit anti-HBA1 (1:1000, ABclonal) at 4 °C overnight. Afterward, the membrane was incubated with HRP-conjugated secondary antibodies at room temperature for 1 h, followed by reacting with the enhanced chemiluminescence substrate. The protein bands were imaged by a chemiluminescence imager (Monna, QuickChemi 5100) and processed with ImageJ software.

### 2.15 Co-immunoprecipitation

Co-immunoprecipitation was performed to evaluate the binding behavior of Hp and Hb proteins. The experiment was performed according to the manufacturer's instructions (Immunoprecipitation Kit with Protein A + G Magnetic Beads, Beyotime). Briefly, the M1-type microglia were transfected with the 5 μg M2-exo@HI for 48 h, and treated with 4 % RBC lysate for 6 h. Then the cells were lysed, and treated with Hp antibody and protein A/G-magnetic beads for the Co-IP assay. Immunoprecipitated proteins were analyzed by WB.

### 2.16 Microglia polarization

To investigate the ability of microglia polarization by M2-exo@HI, in vitro cerebral hemorrhage model was used to simulate hemorrhagic stroke injury. Briefly, BV2 cells (2 × 10^5^ per well) were inoculated in confocal dishes with 4 % RBC lysate [20]. After 6 h, cells were cultured in a normal medium and treated with PBS, 5 μg HI, 20 μg M2-exo, and 25 μg M2-exo@HI (20 μg M2-exo and 5 μg HI), respectively. After 24 h of incubation, cells were labeled with CD163-APC (1:100, Abcam) and CD86-PE (1:100, Abcam) for flow cytometry analysis.

### 2.17 Erythrocyte isolation, treatment, and labeling for phagocytosis assay

Whole blood was collected from C57BL/6 mice into heparinized tubes and centrifuged at 500×*g* for 10 min at 4 °C. The erythrocyte pellet was washed three times with PBS and counted. A total of 1 × 10^9^ RBCs were treated with PBS or 0.75 mM tert-butyl hydroperoxide (t-BHP, Sigma-Aldrich) for 30 min to induce artificial aging through promoting phosphatidylserine exposure and reducing CD47 expression. After treatment, cells were centrifuged and counted again. Apoptotic RBCs were labeled with Annexin V–mCherry (Beyotime) according to the manufacturer's protocol and observed under a fluorescence microscope. For membrane staining, RBCs were incubated with 10 μM 3,3′-dioctadecyloxacarbocyanine perchlorate (DiO) in the dark for 15 min, washed, and centrifuged. Labeling efficiency was assessed by flow cytometry, and cell concentration was adjusted for subsequent phagocytosis assays.

### 2.18 Erythrophagocytosis by M2-exo@HI

BV2 cells were seeded in confocal dishes at a density of 1 × 10^5^ cells per dish and cultured for 24 h. The cells were then treated with PBS, 5 μg HI, 20 μg M2-exo, and 25 μg M2-exo@HI, respectively. After 24 h of incubation, DiO-labeled RBCs were added to the microglia cultures at a 10:1 ratio (RBCs to microglia) and co-cultured for 2 h. Subsequently, the supernatant was removed, and the cells were fixed with 4 % paraformaldehyde. Immunofluorescence staining was performed using a mouse anti-CD11b-PE antibody (1:100, Elabscience), and observed by confocal microscope.

### 2.19 In vitro BBB repair

The bEnd.3 cells were seeded at a density of 2 × 10^5^ cells per well in the upper chamber of transwell and cultured until a confluent monolayer formed. The monolayer was then treated with 4 % RBC lysate. After 6 h of incubation, the medium was replaced with supernatants collected from microglial cultures that had been sequentially treated with 4 % RBC lysate for 6 h, followed by 24 h of treatment with PBS, HI, M2-exo, or M2-exo@HI, respectively. Then the barrier integrity of the bEnd.3 monolayer was assessed by adding FITC-dextran (20 kDa) to the upper chamber and measuring the fluorescence in the lower compartment using a microplate reader.

### 2.20 In vitro neuroprotection

The PC12 cells were seeded in a 24-well plate at a density of 2 × 10^5^ per well and cultured for 24 h. Subsequently, the cells were exposed to 4 % RBC lysate for 6 h, followed by treatment with the conditioned media derived from BV2 cells that had been sequentially incubated with 4 % RBC lysate for 6 h and then with either PBS, HI, M2-exo, or M2-exo@HI for 24 h. After treatment, the PC12 cells were collected and stained using an Annexin V-FITC apoptosis detection kit according to the manufacturer's instructions, followed by analysis with flow cytometry.

### 2.21 ICH model

Male C57BL/6 mice (8–12 weeks, 25–30 g) were purchased from Beijing Huafukang Biotechnology Co., Ltd. Mouse ICH model were induced by intra-striatal injection of bacterial collagenase as previously described [21]. Briefly, mice were anesthetized with ketamine/xylazine mixture by intraperitoneal injection and positioned prone in a stereotactic head frame. A hole with a diameter of ∼1 mm was drilled on the right side of the skull (2.3 mm lateral to the midline, 0.5 mm anterior to the bregma). Then 0.5 μL of 0.0375 U bacterial collagenase (type IV, Sigma-Aldrich, St. Louis, MO) was administered at the same position. An identical surgical procedure with equal volume of saline was injected for the sham-operated group. The cranial burr hole was sealed with bone wax, and the incision was sutured. Body temperature was maintained at 37.0 ± 0.5 °C throughout the procedure. All animal experimental procedures were approved by the Institutional Animal Care and Use Committee of Southwest Jiaotong University (SWJTU-2203-NSFC (007)), and the experiments were conducted in strict accordance with the applicable experimental animal ethics regulations. ICH mice were randomly divided into 3 groups and intravenously injected with 200 μL of PBS, HI (100 μg/kg), M2-exo (400 μg/kg) and M2-exo@HI (M2-exo: 400 μg/kg; HI: 100 μg/kg) at 6 h, 24 h and 48 h post-stroke.

### 2.22 Near-infrared fluorescence imaging

To evaluate the brain-accumulation ability of M2-exo, ICH mice were intravenously injected with the near-infrared fluorescent dye ICG and M2-exo@ICG, respectively. Then mice were anesthetized with 5 % isoflurane and imaged using near-infrared fluorescence in vivo imaging system (IVIS, Caliper Life Sciences, USA) at predetermined time intervals (1 min, 5 min, 10 min, 15min, 2 h, 6 h, 12 h, 24 h). To observe the fluorescence distribution of ICG and M2-exo@ICG in ICH mice, tissues including heart, liver, spleen, lung, kidney, and brain were collected at 24 h post-injection for imaging.

### 2.23 Mechanism of brain targeting and M1 microglia homing

To investigate the mechanism underlying M2-exo accumulation in the ICH region, blocking experiments were performed using an anti-CD206 antibody and RGD peptide. First, M2-exo@ICG was incubated separately with the CD206 antibody or RGD peptide at 37 °C for 30 min. Then, ICH model mice were intravenously injected with M2-exo@ICG, M2-exo@ICG pre-incubated with CD206 antibody (M2-exo@ICG + CD206), or M2-exo@ICG pre-incubated with RGD peptide (M2-exo@ICG + RGD). In vivo fluorescence imaging was conducted using IVIS at predetermined time points (15 min, 2 h, 6 h, 12 h, and 24 h post-injection). Brains were harvested 24 h after injection for ex vivo imaging. To examine the cellular uptake of M2-exo in the hemorrhagic brain, brain tissues were collected from mice treated with M2-exo@RhB, M2-exo@RhB + CD206, or M2-exo@RhB + RGD. The tissues were homogenized and subjected to flow cytometry after staining with FITC-conjugated Iba-1 (1:100, Proteintech), APC-conjugated NeuN (1:100, Proteintech), and APC-conjugated GFAP (1:100, eBioscience).

### 2.24 BBB penetration

The BBB penetration of M2-exo was assessed using a two-photon microscope (A1RMP+, Nikon). A cranial window was created over the hemorrhagic hemisphere in ICH model mice, followed by sequential injection of 100 μL of Evans blue (EB, 2 %) and 100 μL of M2-exo@RhB (1 mg/mL). Z-stack images were acquired immediately, scanning from 200 μm to 500 μm below the cortical surface. Extravasation of both EB and M2-exo@RhB from the impaired cerebral vessels could be clearly observed.

### 2.25 Brian water content

To study the effect of different groups on cerebral edema, the hemorrhagic brains of mice were excised after treatment and weighed to record the wet weight. The brains were then freeze-dried and weighed to record the dry weight. The water content of the brain was calculated as Equation (5).

(5) $$\text{Water}\;\text{content}\;\left(\%\right)=\frac{\text{Wet}\;\text{weight}-\text{Dry}\;\text{weight}\;}{\text{Wet}\;\text{weight}}\times 100\%$$

### 2.26 Hematoxylin and eosin (HE), Nissl, and TUNEL staining

To evaluate the brain injury by immunohistochemistry, mice were sacrificed 3 days after ICH surgery. The brain tissues were collected and cut into 3 μm sections for HE, Nissl and TUNEL staining. The sections were observed under a microscope slide scanner (PANNORAMIC MIDI, 3DHISTECH). The images were analyzed by ImageJ, and the average optical density (AOD) value was calculated by Equation (6).

(6) $$\text{AOD}\;\left(\%\right)=\frac{\text{IOD}\;}{\text{Area}}\times 100\%$$

### 2.27 Behavioral test

To investigate the long-term motor and cognitive functions of ICH mice after treatment, a series of behavioral tests were performed within 28 days. Rotarod test, adhesive test, balance beam test, and Barnes maze test were performed three times a day. The cylinder test was performed once on each test day. The modified neurological severity scores (mNSS) has a total of 18 points, including 6 points for the motor test, 2 points for the sensory test, 6 points for the beam balance test, and 4 points for a reflex loss and abnormal movement [[22], [23], [24]].

#### 2.27.1 Rotarod test

This test was used to assess motor function after stroke. Mice were placed on a rotating rod with accelerating speed from 5 to 20 turns per minute. The mean time to stay on the rod was recorded as the latency to fall. Mice were trained three times a day for 5 days prior to the ICH surgery.

#### 2.27.2 Cylinder test

This test was used to assess forepaw use and rotational asymmetry. Mice were placed into a transparent cylinder (height: 15 cm; diameter: 9 cm), and a video camera was used to record the contact of the forepaw with the cylinder for 10 min. The asymmetry rate was calculated as Equation (7).

(7) $$\text{Asymmetry}\;\left(\%\right)=\frac{L-R\;}{L+R+B}\times 100\%$$

L: the number of left forepaw contacts, R: the number of right forepaw contacts, and B: the number of both contacts.

#### 2.27.3 Adhesive test

This test was used to evaluate the tactile response and sensorimotor function. A 3 × 3 mm^2^-sized patch was attached to the paralyzed forepaw of ICH mice. The time for the mice to successfully remove the patch was recorded.

#### 2.27.4 Morris water maze test

This test was used to assess spatial learning and reference memory ability after a stroke. The maze consisted of a 120-cm diameter circular plastic pool filled with opaque water at 25 ± 2 °C during testing. The pool was divided into four quadrants equally named I, II, III, and IV, respectively. The Morris water maze test lasted for 6 days as previously described. Briefly, mice were given orientation navigation test for 5 consecutive training days (4 trials a day). A hidden circular platform (diameter of 9 cm and a height of 30 cm) was placed under 1 cm of water in the middle of one quadrant (target quadrant) in all trials. For each daily trial, mice were randomly placed at one of the four equally spaced starting locations within the pool, and allowed to sit on the platform for 10 s after climbing onto it successfully. The maximum time allowed per trial was 90 s. If mice failed to locate the platform within 90 s, they were guided to the platform by the experimenter and kept there for 10 s. On the 6th day, for the probe trial, the platform was removed, and mice were allowed to swim freely in the pool for 90 s. The escape latency onto the platform, the time spent in the target quadrant, and the number of platform crossing times were recorded using a video camera and analyzed using the SMART 3.0 behavioral recording system (Panlab, Spain).

### 2.28 Immunofluorescence staining

To perform immunofluorescence staining, mouse brains were separated at day 3 after ICH and made into 20 μm frozen coronal sections. The first antibodies included rabbit anti-IBA-1 antibody (1:100, Abcam), rabbit anti-CD31 (1:200, Abcam), rabbit anti-GFAP (1:100, Abcam), rabbit anti-NeuN (1:150, ABclonal), rabbit anti-HP (1:200, ABclonal), rabbit anti- HBA1 (1:200, ABclonal), rabbit anti-p65 (1:200, Proteintech), rabbit anti-CD163 (1:100, eBioscience), and rabbit anti-CD86 (1:100, Abcam). The secondary antibodies included Alexa Fluor 647-conjugated goat anti-rat IgG (1:1000, Abcam), Alexa Fluor 488-conjugated goat anti-rat IgG (1:1000, Abcam), Alexa Fluor 488-conjugated goat anti-rabbit IgG (1:1000, Servicebio), Alexa Fluor 594-conjugated donkey anti-goat IgG (1:1000, Servicebio), and Alexa Fluor 647-conjugated goat anti-rabbit IgG (1:1000, Servicebio). The staining sections were observed using Ortho fluorescence microscope (Beijing Shiji Kexin Scientific Instrument Co., Ltd).

### 2.29 Evans blue (EB) staining

To evaluate the permeability of BBB in mice, 200 μL of EB (2 %, Sigma-Aldrich) solution was intravenously injected 72 h post ICH. After 2 h, the brains were separated for photographing. To quantify the EB concentration, brains were weighed and homogenized in 4 mL of dimethylformamide. Then the homogenate was incubated at 37 °C for 48 h and centrifuged (10000 rpm, 20 min) to collect the supernatant. The content of EB was determined by measuring the absorbance of the supernatant at 632 nm.

### 2.30 In vivo safety evaluation

To analyze the biocompatibility of exosomes, organs including heart, liver, spleen, lung, kidney, and blood were collected 3 days and 28 days after ICH. HE staining, blood routine, and blood biochemistry tests were performed, respectively. CaseViewer was used for image analysis and processing. To detect the HI plasmid clearance in vivo, we measured HI plasmid levels in major organs (brain, liver, spleen, lung, and kidney) at different time points after a single administration by qPCR.

### 2.31 ELISA test

To detect cytokines and proteins in the microglia supernatant and brain tissues, the ELISA method was performed. ELISA kits included mouse TNF-*α* ELISA kit (Solarbio), mouse IL-10 ELISA kit (Elabscience), mouse Hp ELISA kit (Elabscience), mouse IL-1β ELISA kit (Solarbio), and TGF-β ELISA kit (Solarbio).

### 2.32 Transcriptome analysis

The hemorrhagic brains of mice at 3 days after ICH were separated for RNA extraction and quantified using a NanoDrop 2000 spectrophotometer (Thermo Scientific, USA). RNA integrity was analyzed by Agilent 2100 Bioanalyzer (Agilent Technologies, Santa Clara, CA, USA). The libraries were constructed using TruSeq Stranded mRNA LT Sample Prep Kit (Illumina, San Diego, CA, USA). The transcriptome sequencing and analysis were conducted by Majorbio BioTech Co., Ltd. (Shanghai, China).

### 2.33 qPCR analysis

To detect the expression levels of Hp, IL-10, Cd163, Cxcr4, Stat3, and Igf2 in cells or tissues, the total RNA was extracted from BV2 cells or mouse brains after therapy for qPCR analysis. The extracted RNA was then converted to cDNA by 5X All-In-One RT MasterMix. The relative content of the target gene was detected by QuantStudio TM3 using SsoAdvanced Universal SYBR Green Supermix. The result was analyzed by 2^−ΔΔCt^ method. The following information regarding the primers was obtained:

Hp Forward: GCTATGTGGAGCACTTGGTTC.

Reverses: CCCATTGCTTCTCGTCGTT.

IL-10 Forward: AGGGCACCCAGTCTGAGAACA.

Reverses: CGGCCTTGCTCTTGTTTTCAC.

Cd163 Forward: TACAATGGAGCTTGGGGCAG.

Reverses: TGCCTCGATGGTGTCTTGTC.

Cxcr4 Forward: ACTACACCGAGGAAATGGGCT.

Reverses: CCCACAATGCCAGTTAAGAAGA.

Stat3 Forward: ATTGCCCGGATTGTGGCCCG.

Reverses: CTCCGTCACCACGGCTGCTG.

Igf2 Forward: GGAGGGGAGCTTGTTGACAC.

Reverses: TGGCACAGTATGTCTCCAGG.

GAPDH Forward: CCTCGTCCCGTAGACAAAATG.

Reverses: TGAGGTCAATGAAGGGGTCGT.

CMV Forward: ATGGGCGTGGATAGCGG.

Reverses: CACGCCTACCGCCCAT.

### 2.34 Intracerebroventricular injection for key signaling pathway validation

To validate key signaling pathways, intracerebroventricular injection was performed as previously described [25]. Briefly, a cranial burr hole was made at the following coordinates relative to bregma (0.3 mm posterior, 1.0 mm lateral, and 2.3 mm deep). The needle of a 10-μl Hamilton syringe was carefully inserted into the left ventricle through the burr hole, and infusion was conducted at a rate of 2 μl/min. After completion of the infusion, the needle was left in place for an additional 8 min to ensure adequate distribution and was then slowly withdrawn over 3 min. The Phorbol-12-myristate-13-acetate (PMA) was also dissolved in dimethyl sulfoxide (DMSO) and then injected into the right lateral ventricle by intracerebroventricular administration (0.1 nmol/mice) at 1 h after ICH. Then, the ICH mice were randomly divided into 4 groups and intravenously injected with PBS, HI, M2-exo M2-exo@HI, and M2-exo@HI + PMA at 6 h, 24 h and 48 h post-stroke. On day 3 post-modeling, brain tissue from the hemorrhagic site was collected for Western blot analysis.

### 2.35 Statistical analysis

All experiments were carried out with a minimum of three independent devices for each experimental group (n ≥ 3). Statistical analysis was performed by GraphPad Prism (10.1.2) or genomic software. For comparison of means of samples with normal distribution and homogeneous variance, an unpaired *t*-test was used for two groups. For samples with more than two groups, one-way or two-way ANOVA test was used for multiple comparisons. Data are presented as mean ± SD. For all Figures: ns, not significant; ∗, *p* < 0.05; ∗∗, *p* < 0.01; ∗∗∗, *p* < 0.001.

## 3 Results and discussion

### 3.1 Fabrication and characterization of M2-exo@HI

**Fig. 2:**
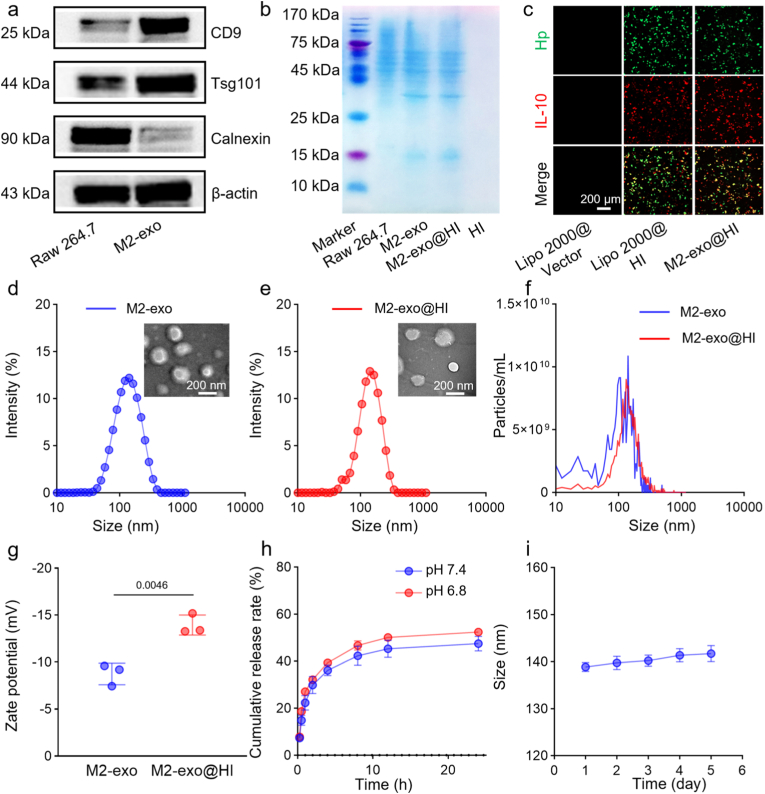
Characterization of M2-exo@HI. (a) Western blot of Tsg101, CD9, and Calnexin expressions in RAW264.7 and M2-exo. (b) Protein bands of RAW264.7, M2-exo, M2-exo@HI, and HI by SDS-PAGE. (c) Fluorescence microscopy images showing Hp-EGFP and IL-10-mCherry expression in 293T cells infected with lipo2000@pBudCE4.1, lipo2000@HI, and M2-exo@HI respectively, after 24 h. (d,e) Representative TEM images and size distribution profiles of M2-exo and M2-exo@HI. (f) Particle number of M2-exo and M2-exo@HI by NTA measurement. (g) Zeta potentials of M2-exo and M2-exo@HI (n = 3). (h) Drug release profiles of M2-exo@HI at pH 6.5 and pH 7.4, respectively (n = 3). (i) Stability evaluation of M2-exo@HI in PBS at 4 °C by monitoring particle size over time (n = 3). Data are presented as mean ± SD.

To prepare M2-exo@HI, RAW264.7 macrophages were first polarized to the M2 phenotype using DEX (Fig. 1a) [26]. Phenotypic conversion was validated through characterization of surface markers and cytokine secretion. Flow cytometry analysis showed dose-dependent upregulation of CD206 expression in macrophages following DEX treatment, with optimal polarization efficiency (over 90 %) achieved at 2 μM concentration (Fig. S1 and S2). At this dose, DEX-treated macrophages exhibited a characteristic M2 cytokine profile, with significantly elevated secretion of anti-inflammatory cytokines (IL-10, IL-4) along with markedly suppressed production of proinflammatory mediators (TNF-α, IL-1β) compared to untreated control and macrophages treated with suboptimal DEX concentrations (0.2–1 μM) (Fig. S3). Importantly, macrophage proliferation was not affected differently at all tested concentrations of DEX, establishing 2 μM as the optimal dose for subsequent experiments (Fig. S4). M2-exo were then isolated from cell supernatants via differential centrifugation. Western blot analysis confirmed the overexpression of exosomal markers (CD9 and Tsg101) and decreased expression of the endoplasmic reticulum marker Calnexin, verifying the purity of exosomes derived from both RAW264.7 (Fig. 2a; Fig. S5) [27]. The sodium dodecyl sulfate-polyacrylamide gel electrophoresis (SDS-PAGE) showed nearly identical protein profiles between M2-exo and M2-exo@HI (Fig. 2b), indicating maintained exosomal integrity following cargo loading. Concurrently, we engineered the dual-expression plasmid pBudCE4.1-Hp/IL-10 for simultaneous production of Hp and IL-10 (Fig. S6). At 24 h post-transfection, fluorescence microscopy revealed obvious expression of both Hp-EGFP and IL-10-mCherry in 293T cells (Fig. 2c), thereby verifying the successful construction of the functional plasmid. Quantitative analysis of fluorescence intensity further confirmed efficient gene expression (Fig. S7). These results suggest that M2-exo is a promising plasmid delivery system capable of mediating efficient gene expression.

The HI plasmids were subsequently loaded into M2-exo via electroporation to construct M2-exo@HI. A voltage of 250 V was selected as the experimental parameter due to its optimal loading efficiency (25.0 %), plasmid integrity, and expression performance (Fig. S8). TEM revealed that M2-exo@HI retained the characteristic cup-shaped morphology of exosomes (Fig. 2d and e). DLS and NTA indicated mean particle diameters of 127.6 ± 11.97 nm for M2-exo and 131.7 ± 15.31 nm for M2-exo@HI, with a slight reduction in particle concentration from 2.0 × 10^11^ particles/mL to 1.8 × 10^11^ particles/mL post-electroporation (Fig. 2f). Zeta potential measurements showed a significant shift from −8.8 ± 1.1 mV (M2-exo) to −14.0 ± 1.1 mV (M2-exo@HI) (Fig. 2g), confirming successful plasmid encapsulation. This shift likely results form charge masking by anionic DNA and electroporation-induced phosphatidylserine externalization on the exosomal membrane [28,29]. M2-exo@HI exhibited pH-responsive release kinetics, with accelerated HI release at pH 6.8 compared to pH 7.4 (Fig. 2h). This behavior can be attributed to acid-triggered conformational changes in fusogenic proteins, promoting exosomal membrane deformation and cargo release [30,31]. Additionally, M2-exo@HI exhibited excellent stability in PBS, maintaining consistent size and zeta potential over 5 days at 4 °C (Fig. 2i; Fig. S9). In vitro biocompatibility assessment demonstrated excellent cellular tolerance, with >85 % viability in BV2 microglial cells and ECs following M2-exo@HI treatment (Fig. S10a). Hemocompatibility studies showed negligible coagulation effects and hemolysis rate <5 % (Fig. S10b and c), collectively establishing the platform's suitability for biomedical applications.

### 3.2 In vitro phagocytosis investigation

**Fig. 3:**
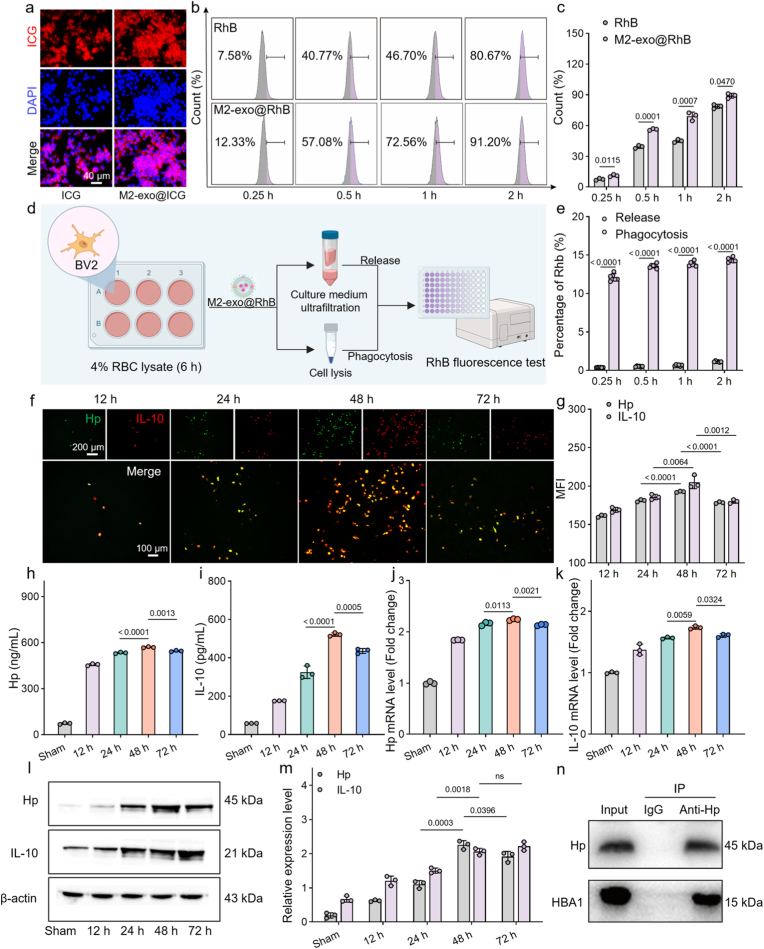
Validation of M2-exo targeting, Hp/IL-10 transfection expression, and Hp/Hb binding. (a) Fluorescence imaging showing cellular uptake of ICG and M2-exo@ICG by M1 microglia. (b,c) Flow cytometry and corresponding quantification of RhB and M2-exo@RhB internalized by M1 microglia (n = 3). (d,e) Schematic illustration and quantitative analysis of the in vitro phagocytosis-release kinetics of M2-exo@RhB in BV2 under ICH-mimicking stimulation (n = 6). (f) Fluorescence images showing Hp and IL-10 expression in M1 microglia treated with M2-exo@HI for 12, 24, 48, 72 h. (g) Mean fluorescence intensity (MFI) quantification of Hp and IL-10 expression (n = 3). (h,i) ELISA measurements of secreted Hp and IL-10 protein levels (n = 3). (j,k) qPCR analysis of relative Hp and IL-10 mRNA expression (n = 3). (l) Western blot detection of Hp and IL-10 protein expression. (m) Densitometric quantification of Hp and IL-10 protein levels from Western blot (n = 3). (n) Co-immunoprecipitation assay confirming the formation of Hp-Hb complex. Data are presented as mean ± SD. Statistical significance was calculated by unpaired Student's *t*-test (c and e), and one-way ANOVA with Tukey's multiple comparisons test (g-k and m).

To investigate the inflammatory targeting capacity of M2-exo, microglia were first treated with LPS to be converted to M1 phenotype (Fig. S11 and S12). Then ICG and RhB were loaded into M2-exo to obtain M2-exo@ICG and M2-exo@RhB for cellular uptake study. Fluorescence microscopy (Fig. 3a; Fig. S13) demonstrated time-dependent cellular accumulation after treating M1 microglia. Notably, M2-exo@ICG exhibited significantly enhanced fluorescence intensity compared to free ICG at all time points. Consistent with fluorescence microscopy results, the quantitative flow cytometry analysis further confirmed a significantly higher uptake of M2-exo in M1 microglia (Fig. 3b and c). The superior cellular uptake observed for M2-exo@ICG or M2-exo@RhB strongly suggests active, ligand-receptor mediated targeting to M1 microglia, resulting in improved phagocytic uptake efficiency [32].

Additionally, to determine whether the payload was primarily released into the extracellular environment before cellular uptake or initially internalized and retained by microglia, we conducted an “uptake-release” assay under ICH-mimicking conditions. As shown in Fig. 3d and e, microglia exhibited rapid and dominant uptake, with a phagocytosis rate reaching 12.11 % at 0.25 h and slightly increasing to 14.37 % by 2 h. In contrast, extracellular RhB release remained minimal throughout the observation period, accounting for ≤1 % between 0.25–1 h and only 1.14 % at 2 h. These findings indicate that under ICH-like stimulation, M2-exo are substantially internalized by BV2 cells at early time points, with uptake levels markedly exceeding detectable extracellular release. This supports an “uptake-first, release-later” mechanism rather than significant payload release prior to microglial entry.

### 3.3 Verification of Hp and IL-10 expression in microglia and Hp/Hb binding

Following intracellular delivery of HI plasmids via M2-exo@HI, fluorescence imaging of M1 microglia revealed robust and colocalized expression of Hp-EGFP (green) and IL-10-mCherry (red), reaching maximal intensity by 48 h (Fig. 3f and g). This temporal expression pattern was further corroborated by quantitative ELISA measurement, which demonstrated progressive accumulation of both therapeutic proteins in culture supernatants within 48 h (Fig. 3h and i). To evaluate the potential of M2-exo as plasmid carriers, we measured the expression of Hp and IL-10 proteins by microglia following transfection with different plasmid doses (1, 2.5, 5, 10, and 15 μg). As shown in Fig. S14, the M2-exo@HI group achieved the highest protein expression with a plasmid dose of 5 μg, whereas the Lipo 2000@HI group required 15 μg to reach its maximum expression. Notably, at equivalent plasmid doses, M2-exo consistently elicited higher expression levels of both Hp and IL-10 compared to Lipo 2000. These results underscore the superior delivery efficiency of M2-exos, which enable strong protein expression at lower plasmid doses than conventional transfection reagents. Transcriptional and translational kinetics showed progressive expression of Hp mRNA (Fig. 3j) and corresponding proteins (Fig. 3l and m) in microglia over time. The quantitative protein expression profiles correlated directly with fluorescence intensities, validating sustained transgene expression following M2-*exo*-mediated delivery. Notably, qPCR analysis revealed that IL-10 mRNA expression peaked at 48 h (Fig. 3k), whereas Western blot detected maximal IL-10 protein levels at 72 h (Fig. 3l; Fig. S15). This temporal discrepancy is consistent with the known decoupling of mRNA and protein dynamics, which can arise from differences in transcript stability, translation rates, and protein degradation [33]. In macrophages, IL-10 expression is subject to multi-level temporal regulation: early transcriptional activation is followed by interferon-mediated translational control, and inflammatory polarization further modifies post-transcriptional and translational programs, ultimately resulting in delayed protein accumulation relative to mRNA [[34], [35], [36]]. To determine whether the expressed Hp binds extracellular Hb, we performed co-immunoprecipitation assays using microglial lysates. WB confirmed the formation of Hp-Hb complexes (Fig. 3n), demonstrating functional interaction between plasmid-derived Hp and cell-associated Hb.

### 3.4 In vitro microglia polarization and RBC phagocytosis

**Fig. 4:**
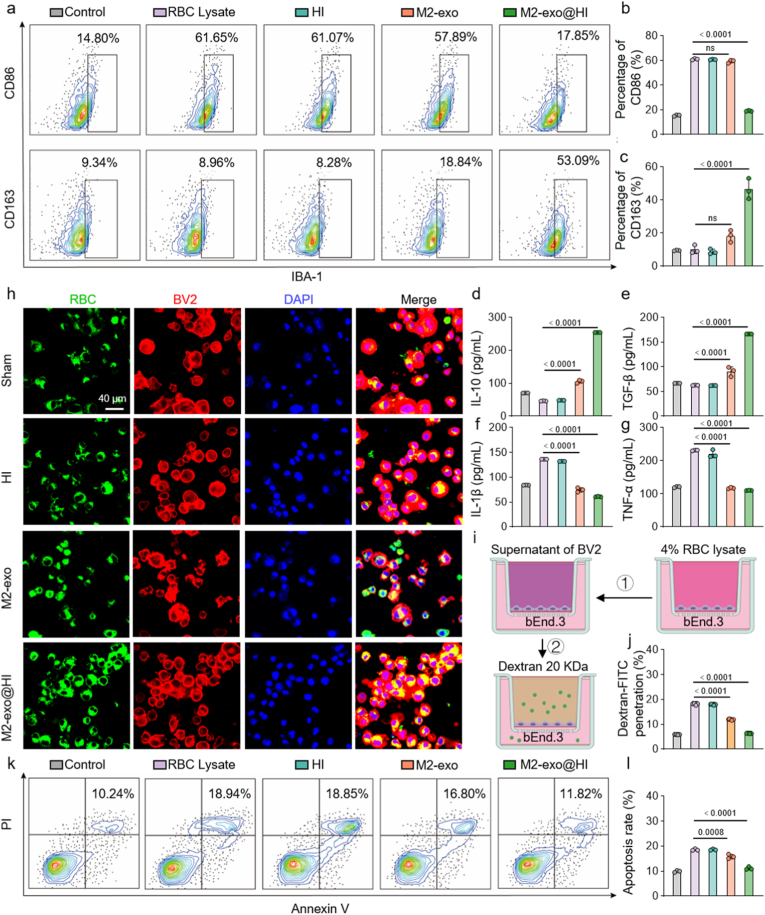
M2-exo@HI promotes in vitro microglia polarization, BBB repair and neuroprotection. (a) Flow cytometry analysis of M1-type (CD86^+^) and M2-type microglia (CD163^+^) following treatment with different formulations. (b,c) Percentages of CD86^+^ and CD163^+^ microglia populations (n = 3). (d–g) The cytokine levels of IL-10, TGF-β, TNF-α, and IL-1β in treated microglia (n = 3). (h) Fluorescence microscopy images showing erythrophagocytosis by microglia across treatment groups. (i) Schematic of the in vitro BBB model assessing FITC-dextran permeability using a transwell assay. (j) Quantitative analysis of FITC-dextran penetration (n = 7). (k) Flow cytometry analysis of neuronal apoptosis across treatments (n = 3). (l) Quantitative analysis of neuronal apoptosis (n = 3). Data are presented as mean ± SD. Statistical significance was tested by one-way ANOVA with Tukey's multiple comparisons test.

To investigate the effect of M2-exo@HI on microglia polarization, we first established an in vitro ICH model by incubating BV2 cells with 4 % RBCs hemolysate. While M2-exo alone showed negligible ability to promote M2 microglial polarization, treatment with M2-exo@HI significantly increased the CD163^+^ microglia population to 53.09 %, representing a 5.9-fold increase compared to ICH group (Fig. 4a–c). Crucially, M2-exo@HI restored the pathological imbalance in inflammatory mediators, reducing pro-inflammatory cytokines (IL-1β: 53.6 %; TNF-α: 49.9 %) while upregulating anti-inflammatory factors (IL-10: +80.9 %; TGF-β: +62.6 %) relative to the HI plasmid group (Fig. 4d–g). These results demonstrate the potent anti-inflammatory efficacy of M2-exo@HI, mediated through coordinated cytokine reprogramming.

Next, the erythrophagocytic potential of microglia following M2-exo@HI treatment were studied. Prior to co-culture, RBCs were artificially aged using tert-butylhydroperperoxide (t-BHP) to induce oxidative alterations mimicking pathological RBCs senescence (Fig. S16a and b) [37]. Following labeling with DiO fluorescent dye **(**Fig. S17**)**, these RBCs were co-cultured with treated BV2 cells for 2 h. Confocal imaging revealed significantly enhanced erythrophagocytosis activity in M2-exo@HI-treated microglia compared to other groups (Fig. 4h). Our findings established that M2-exo@HI not only modulates inflammatory responses but also augments the critical hemoglobin clearance pathway, representing a dual therapeutic mechanism for hemorrhagic conditions.

### 3.5 In vitro BBB leakage repair and neuroprotection

To explore the effect of M2-exo@HI on BBB integrity under ICH conditions, we employed FITC-dextran (20 kDa) permeability assay using bEnd.3 monolayers (Fig. 4i). The ICH model induced severe barrier disruption, manifesting as a 3.1-fold increase in FITC-dextran translocation compared to sham control (Fig. 4j). M2-exo@HI treatment effectively restored BBB integrity, reducing paracellular leakage by 65.3 %. To investigate the neuroprotective effect of M2-exo@HI, neuronal cells were cultured in conditioned medium obtained from microglial cells that had been sequentially treated with 4 % RBC lysate and M2-exo@HI, and apoptosis was subsequently assessed (Fig. S18). Flow cytometry analysis of PC12 neuronal apoptosis revealed that M2-exo@HI reduced neuronal apoptosis by 39.9 % versus ICH controls (Fig. 4k and l). These position M2-exo@HI as a promising therapeutic candidate capable of addressing both vascular and neuronal pathologies in ICH.

### 3.6 Brain targeting of M2-exo and in vivo expression of Hp and IL-10 plasmid

**Fig. 5:**
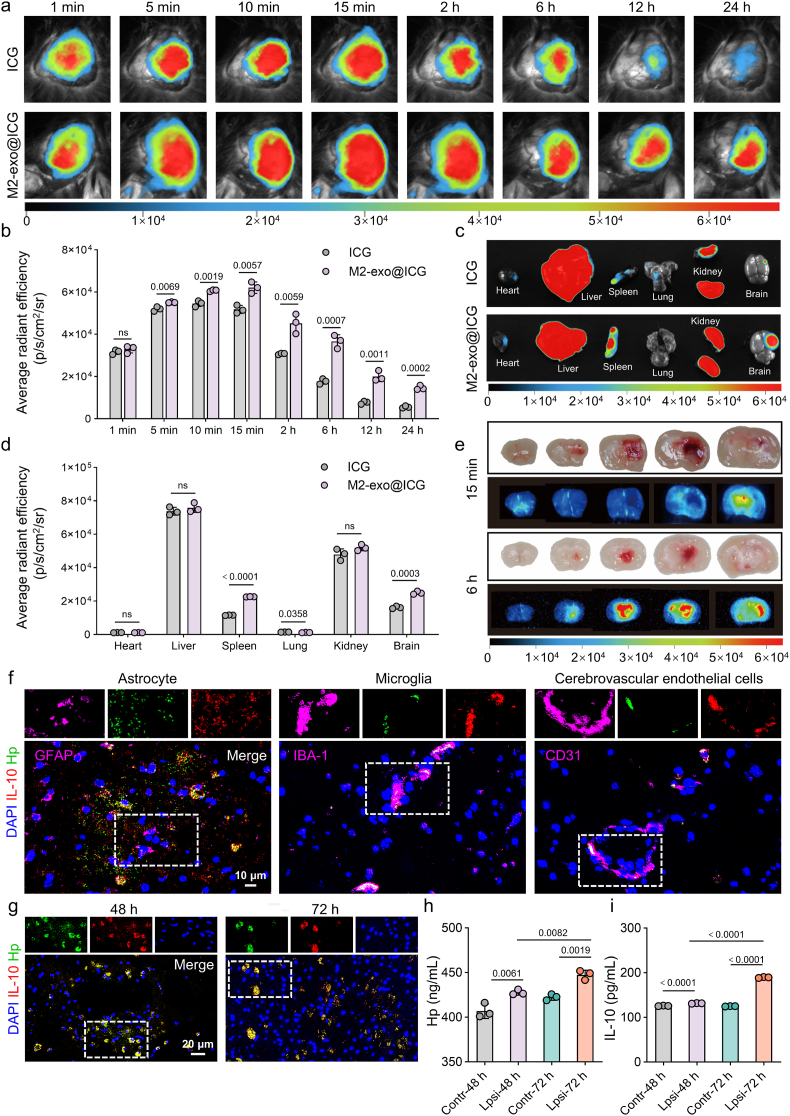
Targeted delivery and therapeutic gene expression of M2-exo@HI in hemorrhagic brain. (a) In vivo near-infrared fluorescence imaging showing ICG and M2-exo@ICG in mouse brains at various time points post-injection. (b) Average radiation efficiency of ICG in different treatment groups (n = 3). (c) Ex vivo fluorescence imaging of major organs harvested 24 h post-injection. (d) Average radiation efficiency of ICG in different in mouse tissues (n = 3). (e) Time-dependent accumulation of M2-exo@ICG at hematoma regions. (f) Immunofluorescence staining showing co-localization of Hp/IL-10 with astrocytes, microglia, and endothelial cells. (g) Temporal expression profiles of Hp and IL-10 in brain tissues. (h,i) ELISA quantification of Hp and IL-10 protein levels in brain homogenates (n = 3). Data are presented as mean ± SD. Statistical significance was calculated by unpaired Student's *t*-test (b,d), and one-way ANOVA with Tukey's multiple comparisons test (h,i).

The therapeutic efficacy of M2-exo@HI relies critically on its enrichment capacity in hemorrhagic brain tissue. To validate this, M2-exo@ICG were intravenously administered to ICH mice and performed serial near-infrared fluorescence imaging. Compared to free ICG control, M2-exo@ICG exhibited significantly stronger fluorescence signals in the hemorrhagic hemisphere at 5 min, 10 min, 15 min, 2 h, 6 h, 12 h, and 24 h post-injection (Fig. 5a). This enhanced accumulation can be attributed to both passive and active transport processes. On one hand, the BBB becomes leaky due to tight junction disruption post-ICH, allowing exosomes to extravasate passively [38]. On the other hand, exosome surface proteins (e.g., transferrin receptor) bind to endothelial receptors, triggering vesicular uptake and transcytosis [39]. Quantitative analysis revealed that the mean radiant efficiency in M2-exo@ICG group was 1.06-, 1.11-, 1.20-, 1.47-, 2.07-, 2.61-, and 2.63-fold than in ICG control at the respective time points (Fig. 5b). Ex vivo imaging at 24 h further confirmed the preferential accumulation of M2-exo@ICG in hemorrhagic brain (Fig. 5c and d). Additionally, brain section analysis revealed targeted delivery to hematoma regions, with peak fluorescence intensity observed at 6 h (Fig. 5e).

To evaluate the preferential uptake of M2-exo by brain cells under hemorrhagic conditions, M2-exo@RhB was administered to ICH mice, and brain tissues were subsequently analyzed by flow cytometry. M2-exo was predominantly internalized by microglia, with minimal uptake observed in astrocytes and neurons (Fig. S19), indicating a strong microglial bias. Pre-incubation of M2-exo@RhB with an anti-CD206 antibody modestly reduced microglial uptake (from 29.54 % to 26.27 %), suggesting that CD206 contributes partially, but is not solely responsible for the internalization process. In contrast, pre-incubation with RGD peptide significantly decreased uptake, demonstrating that integrin-mediated interactions play a dominant role in M2-exo entry into the hemorrhagic brain. These findings were further supported by in vivo imaging, which showed that RGD pretreatment impaired M2-exo@RhB retention in the hemorrhagic hemisphere (Fig. S20). Moreover, two-photon microscopy visualized M2-exo@RhB adhering to injured microvessels and extravasating into peri-hematomal tissue (Fig. S21). Together, these results indicate that the targeting of M2-exo to the hemorrhagic brain involves multiple surface-associated mechanisms, with integrin-dependent interactions at the injured endothelium playing a major role, while CD206-associated components contribute partially to microglial uptake [15].

To assess the cellular specificity of Hp and IL-10 expression in hemorrhagic brain tissue, we performed the immunofluorescence co-localization analysis. Tissue sections were immunostained for neuron (NeuN^+^), astrocytes (GFAP^+^), microglial (IBA-1^+^), and endothelial (CD31^+^) markers. Strikingly, microglia exhibited significantly higher co-localization with Hp-EGFP and IL-10-mCherry signals compared to astrocytes, neuron, and endothelial cells (Fig. 5f; Fig. S22), indicating preferential uptake and functional transfection of M2-exo@HI in microglia. Besides, substantial increase in Hp and IL-10 expression was found at 72 h versus 48 h post-injection (Fig. 5g), suggesting sustained transgene activity. Consistently, ELISA quantification demonstrated progressively elevated therapeutic protein levels over time, with significantly higher concentrations in ipsilateral (hemorrhagic brain) than in the contralateral hemisphere (Fig. 5h and i). The delayed yet enhanced in vivo expression profile, compared to earlier transgene activation in vitro (Fig. 3f–m), likely due to the complex biological barriers, reduced transfection efficiency, and immune microenvironmental influences present in the intact organism [40,41]. These findings collectively demonstrate the targeted accumulation of M2-exo@HI at hematoma sites and its efficient transfection of microglia, resulting in sustained therapeutic protein production.

### 3.7 In vivo therapeutic effects on ICH model

**Fig. 6:**
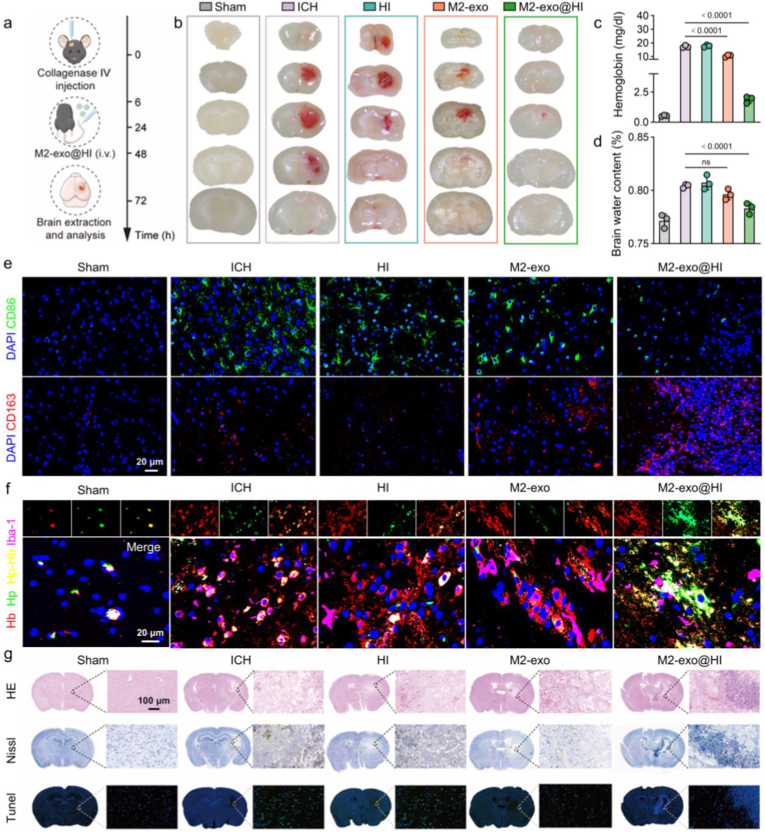
In vivo therapeutic effects of M2-exo@HI in hemorrhagic stroke. (a) The schematic diagram illustrates the construction of mouse cerebral hemorrhage model and treatment regimens. (b) Digital photos showing cerebral hematoma of ICH mice in different groups. (c) Quantitative measurements of hemoglobin concentration in different groups (n = 3). (d) Cerebral edema quantification by brain water content measurements (n = 3). (e) CLSM images showing M1 microglia (CD86^+^, green) and M2 microglia (CD163^+^, red) in different groups. Nucleus were stained with DAPI (blue). (f) Immunofluorescence staining showing co-localization of Hb/Hp with microglia in different groups. (g) Representative images of HE, Nissl, and TUNEL staining. Data are presented as mean ± SD. Statistical significance was tested by one-way ANOVA with Tukey's multiple comparisons test.

The therapeutic efficacy of M2-exo@HI was first assessed by evaluating hematoma resolution (Fig. 6a). A dose-response study revealed a clear improvement in efficacy as the dose increased from 25 μg/kg to 200 μg/kg, with the 100 μg/kg dose chosen for its maximal therapeutic benefit and minimal side effects, which we determined from the extent of brain hemorrhage reduction and hemoglobin levels measured (Fig. S23). M2-exo@HI therapy leads to a significant reduction in hematoma volume compared to other hemorrhagic groups (Fig. 6b). Consistent with this phenomenon, the hemoglobin content in brain tissue was markedly decreased in M2-exo@HI-treated mice, resulting in 89.1 % and 89.4 % reduction in contrast to ICH group and HI group, respectively (Fig. 6c). To investigate the neuroprotective effects, the cerebral edema was measured using dry-wet weight analysis. The ipsilateral hemispheres of M2-exo@HI-treated mice exhibited significantly lower water content (77.4 %) compared to ICH controls (80.5 %) (Fig. 6d), demonstrating effective mitigation of secondary injury.

Immunofluorescence analysis of brain sections revealed distinct microglial polarization patterns following ICH induction. In sham-operated control, microglia remained predominantly quiescent (M0 state) while exhibiting minimal expression of both CD86^+^ (M1 marker) and CD163^+^ (M2 marker) (Fig. 6e). ICH injury triggered significant microglial activation, with a marked increase in CD86^+^ cells and limited CD163^+^ expression, indicating robust M1 polarization. M2-exo@HI treatment effectively reversed this pro-inflammatory shift, reducing CD86^+^ microglia while increasing CD163^+^ populations compared to untreated ICH mice, demonstrating successful microglial repolarization toward the protective M2 phenotype. ELISA quantification of inflammatory mediators in hemorrhagic brains further validated this shift. M2-exo@HI administration reduced M1-associated TNF-α and IL-1β levels by 26.3 % and 57.9 % versus ICH group. Conversely, treatment elevated M2-associated IL-10 and TGF-β by 4.2-, 1.5-fold (Fig. S24). These molecular changes confirm the establishment of an anti-inflammatory microenvironment following M2-exo@HI treatment.

To further examine the cellular uptake of the Hp-Hb complex in vivo, immunofluorescence staining was performed on brain sections from different experimental groups, using triple labeling for Hb (red), Hp (green), and the microglial marker Iba-1 (purple). As shown in Fig. 6f, pronounced colocalization of Hb and Hp within Iba-1^+^ cells was observed in the M2-exo@HI group, yielding strong triple-positive (Hb^+^Hp^+^Iba-1^+^) signals. These results indicate that Hp-bound Hb is actively internalized by microglia following M2-exo@HI treatment. Together, these in vivo findings corroborate our earlier in vitro data and provide direct histological evidence for the microglia-mediated clearance of Hb in the hemorrhagic brain facilitated by M2-exo@HI.

Histopathological evaluation through hematoxylin-eosin (HE) staining demonstrated that M2-exo@HI treatment significantly attenuated neuronal loss and reduced hemorrhagic areas compared to both ICH and HI groups. Nissl staining confirmed enhanced neuronal preservation in cortical regions of M2-exo@HI-treated mice, while TUNEL analysis revealed a substantial reduction in apoptotic cells relative to other hemorrhagic groups (Fig. 6g; Fig. S25). These neuroprotective effects were complemented by significant improvements in vascular integrity. EB extravasation assays showed severe BBB disruption in ICH mice, with extensive parenchymal dye infiltration contrasting sharply with the minimal leakage observed in sham control (Fig. S26a). Treatment with M2-exo@HI markedly vascular permeability, showing a 55 % decrease in EB extravasation compared to ICH controls (Fig. S26b). In contrast, the therapeutic effect of the HI group was significantly limited, and there was no statistically significant difference in dye leakage rate compared to the untreated injury control group.

### 3.8 M2-exo@HI improves long-term neurological functional outcome

To assess long-term functional recovery, ICH mice were subjected to a comprehensive behavioral testing from pre-surgery through post-operative day 28. Serial sensorimotor evaluations including rotarod performance, cylinder test, adhesive removal task, beam balance assessment, along with mNSS, revealed persistent neurological deficits in ICH and HI groups throughout the 28-day observation period (Fig. S27). Notably, M2-exo@HI-treated mice exhibited significantly accelerated and sustained functional recovery, achieving near-complete resolution of neurological impairments by the final assessment time point. Cognitive evaluation through Morris water maze at day 28 post-ICH revealed substantial spatial learning deficits in untreated ICH mice, manifested as significantly prolonged escape latency, extended path length, and increased navigation errors compared to sham group (Fig. S28). In contrast, M2-exo@HI administration significantly mitigated these cognitive deficits, as evidenced by shorter time and fewer errors in finding the escape hole. Collectively, these behavioral outcomes provide compelling evidence that M2-exo@HI treatment potently enhanced both sensorimotor and cognitive recovery following hemorrhagic stroke.

### 3.9 Transcriptomic gene analysis

**Fig. 7:**
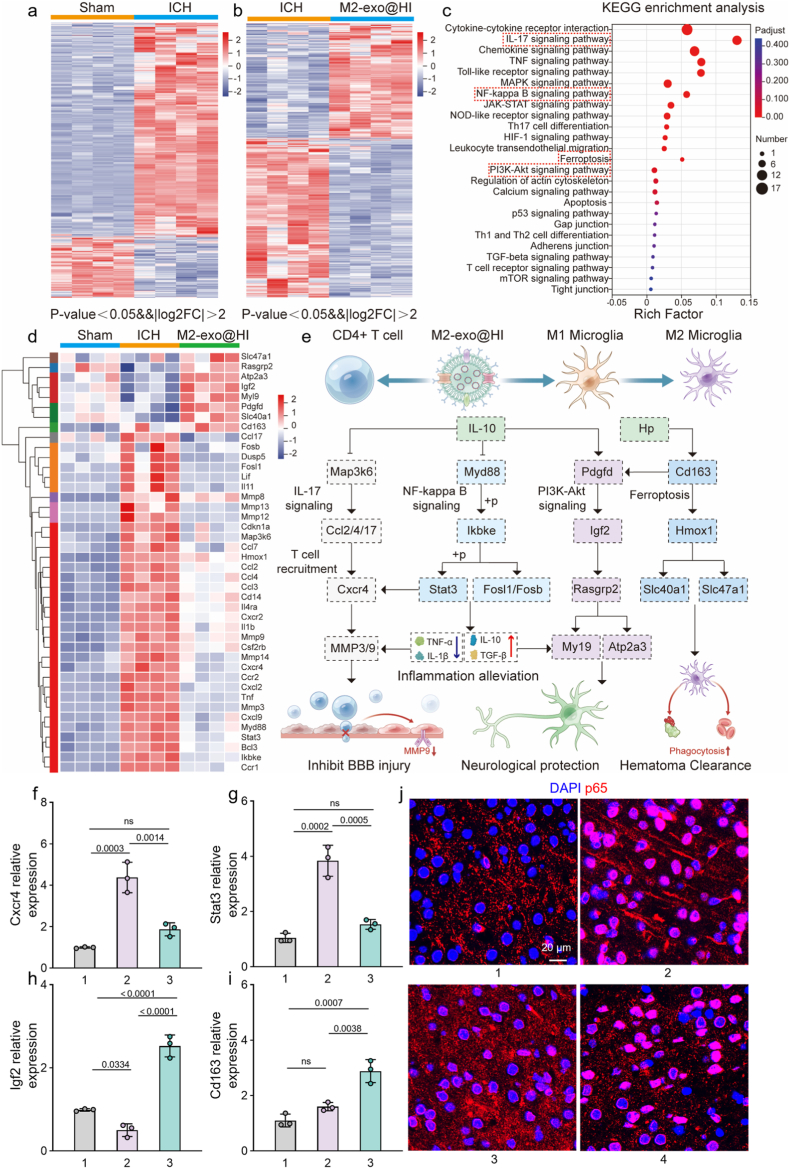
Transcriptomic analysis reveals molecular mechanisms of M2-exo@HI therapy in ICH recovery. (a,b) Heatmap of differentially expressed genes between sham versus ICH and ICH versus M2-exo@HI groups. (c) KEGG enrichment analysis showing significantly modulated pathways following M2-exo@HI treatment. (d) Heatmap of selected genes involved in iron metabolism, inflammation, and tissue repair. (e) Schematic illustration of M2-exo@HI action through coordinated modulation of ferroptosis, NF-κB, and PI3K-Akt pathways. (f–i) qPCR measurement of Cxcr4, Stat3, Igf2 and Cd163 in different groups (n = 3). (j) Representative immunofluorescence images showing p65 protein (red) in the peri-hematomal region of mouse brains. Nucleus were stained with DAPI (blue). 1, Sham; 2, ICH; 3, M2-exo@HI; 4, M2-exo@HI + PMA. Data are presented as mean ± SD. Statistical significance was tested by one-way ANOVA with Tukey's multiple comparisons test.

To elucidate the molecular mechanisms underlying the synergistic anti-inflammatory, hematoma-resolving, and neuroprotective effects of M2-exo@HI, whole-transcriptome profiling was performed. Principal component analysis (PCA) revealed distinct clustering patterns among sham, ICH, and M2-exo@HI groups (Fig. S29), illustrating treatment-specific transcriptomic reprogramming. Differential expression analysis identified 1758 significantly dysregulated genes post-ICH (1396 upregulated and 362 downregulated). Notably, M2-exo@HI treatment modulated 1117 genes in contrast to ICH controls (482 upregulated and 635 downregulated) (Fig. 7a and b). Kyoto Encyclopedia of Genes and Genomes (KEGG) enrichment analysis of these differentially expressed genes highlighted four core therapeutic pathways, including IL-17, NF-κB, PI3K-Akt, and ferroptosis signaling pathways (Fig. 7c). These pathways collectively regulate immune cell infiltration, inflammatory cascades, and cell survival decisions following hemorrhagic injury [[42], [43], [44]]. Further heatmap analysis of differentially expressed genes related to stroke pathology indicated that M2-exo@HI therapy upregulated critical genes involved in iron homeostasis and hemoglobin clearance (Cd163, Slc40a1, Slc47a1) [45,46]. enhanced pro-repair factors mediating angiogenesis and tissue remodeling (Pdgfd, Igf2, Rasgrp2, Atp2a3) [47], while simultaneously suppressing pro-inflammatory mediators (Myd88, Ikbke, Stat3) [48], chemokines (Ccl2/4/7, Cxcr4) [49], and matrix-degrading enzymes (MMP3/9) associated with BBB disruption (Fig. 7d) [50]. The integrated therapeutic mechanism was probably coordinated through Hp/IL-10 co-expression, which orchestrated iron homeostasis restoration via ferroptosis pathway modulation, inflammatory microenvironment remodeling through IL-17/NF-κB inhibition, and enhanced phagocytic clearance coupled with controlled tissue repair via PI3K-Akt signaling (Fig. 7e). This multifaceted action ultimately promotes hematoma resolution, provides neuroprotection, and facilitates BBB restoration. qPCR validation confirmed the transcriptomic findings, with significant upregulation of 1.8-fold of Cd163 and 5.1-fold of Igf2 while downregulation of 57.2 % of Cxcr4 and 60.1 % of Stat3 when comparing M2-exo@HI group with ICH controls (Fig. 7f–i). To validate the key pathway identified through transcriptomic analysis, we performed an intervention experiment using PMA, a known activator of NF-κB signaling [25]. As shown in Fig. 7j and Fig. S30, PMA treatment substantially counteracted the effects of M2-exo@HI and strongly activated NF-κB signaling, as indicated by increased p65 nuclear translocation. These results offer direct functional evidence that the therapeutic benefits of M2-exo@HI are primarily mediated through modulation of NF-κB and associated signaling pathways. To further evaluate the inhibitory effect of M2-exo@HI treatment on CD4^+^ T cell infiltration across the blood-brain barrier, cells were isolated from the peri-hematoma region after 72 h of treatment and analyzed using flow cytometry. The results demonstrated an 84.7 % reduction in the CD4^+^ T cell population in the M2-exo@HI group compared to the ICH control group (Fig. S31).

### 3.10 Toxicity evaluation and clinical translation

To assess the biocompatibility of M2-exo@HI in vivo, we conducted systemic toxicity assessments through histopathological and hematological analyses. Histological examination via HE staining showed negligible pathological abnormalities in major organs (heart, liver, spleen, lung, and kidney) across treatment groups (Fig. S32). Blood routine test revealed no significant differences between M2-exo@HI-treated mice and normal mice, with all hematological parameters (including white blood cell count, red blood cell count, hemoglobin concentration, and platelet count et al.) and and key markers of liver and kidney function (e.g., Ure, Cre, ALT) remaining within normal physiological ranges (Fig. S33). This favorable biosafety profile can be attributed to the targeted delivery of exosomal encapsulation, which effectively confines transgene expression to recipient microglia while preventing plasmid-associated toxicity in peripheral tissues.

To assess the in vivo clearance of the HI plasmid, plasmid copy numbers were quantified in major organs at multiple time points after a single administration. Genomic DNA was extracted from the brain, liver, spleen, lung, and kidney before injection, as well as at 24 h, 72 h, and 1 week post-injection. HI plasmid levels were measured by qPCR using plasmid-specific primers. As shown in Fig. S34, HI copy numbers in the liver and kidney increased transiently at 24–72 h after administration, consistent with the expected initial biodistribution of the formulation, but declined markedly by 1 week. These results indicate that under the dosing regimen used in this study, the HI plasmid does not persist at high levels in vivo and exhibits a self-limited exposure profile. To further evaluate the long-term fate and clearance of M2-exo@HI, in vivo imaging was performed, which revealed no long-term accumulation in major organs. The progressive signal decay observed over 7 days further supports the favorable biosafety profile of M2-exo@HI (Fig. S35).

While the present study demonstrates the favorable biosafety and therapeutic potential of M2-exo@HI in preclinical models, several key challenges must be addressed before clinical translation can be realized. First, the large-scale production of exosome-based nanotherapeutics remains a significant bottleneck [51]. Second, quality control and standardization are crucial for the clinical development of M2-exo@HI [52]. Third, storage stability poses another major translational challenge [53]. Finally, the in vivo metabolic pathways and long-term fate of M2-exo@HI require further investigation [54]. Comprehensive pharmacokinetic, biodistribution, and long-term toxicity studies are therefore essential to fully elucidate the safety profile of M2-exo@HI and support its eventual clinical application.

## 4 Conclusion

In summary, we developed an M2 macrophage-derived exosomal platform (M2-exo@HI) for targeted co-delivery of Hp and IL-10 plasmids to treat intracerebral hemorrhage. This biomimetic nanoplatform harnesses the natural inflammatory tropism of M2-exo to achieve efficient BBB penetration and precise targeting of hemorrhagic lesions, where it demonstrates selective internalization by M1-polarized microglia. The therapeutic efficacy stems from a synergistic dual mechanism whereby secreted Hp effectively clears neurotoxic hemoglobin while IL-10 reprograms microglia toward the protective M2 phenotype, collectively resolving hematoma and inhibiting neuroinflammation. Our results demonstrate significant restoration of neurological function and BBB integrity. This work establishes M2-exo@HI as a therapeutically viable, multi-targeted strategy for hemorrhagic stroke that concurrently addresses inflammation, iron toxicity and neural damage.

## CRediT authorship contribution statement

**Ke Wang:** Writing – original draft, Investigation, Data curation, Conceptualization. **Jin Li:** Software, Resources, Methodology. **Meng He:** Investigation, Funding acquisition. **Liping Wang:** Data curation. **Xing Guo:** Writing – review & editing, Visualization, Funding acquisition, Conceptualization. **Shaobing Zhou:** Supervision, Funding acquisition.

## Ethics approval and consent to participate

This study involved animal experiments only and did not include any human participants. No case-specific details, personal data, or images of patients or any other individuals were collected, used, or reported. All animal experimental procedures were approved by the Institutional Animal Care and Use Committee of Southwest Jiaotong University (SWJTU-2203-NSFC (007)).

## Declaration of competing interest

The authors declare that they have no known competing financial interests or personal relationships that could have appeared to influence the work reported in this paper.
