# Supplementary material for: M2 macrophage-derived exosomes delivering haptoglobin and interleukin-10 plasmids for synergistic therapy of intracerebral hemorrhage
Source: Bioact Mater. 2026 Feb 3;60:510–26. doi: 10.1016/j.bioactmat.2026.01.047 (PMC12887785; doi:10.1016/j.bioactmat.2026.01.047)
Supplement: Multimedia component 1 [file mmc1.docx]

Supporting Information

**M2 macrophage-derived exosomes delivering haptoglobin and interleukin-10 plasmids for synergistic therapy of intracerebral hemorrhage**

**Supplementary Figure Captions:**

**Fig. S1**. Representative flow cytometry plot of the M2-type macrophage (labeled with CD206^+^) and M1-type macrophage (labeled with CD86^+^) among RAW264.7 with dexamethasone in different concentrations.

**Fig. S2.** Percentages of CD86^+^ and CD206^+^ cells in different groups.

**Fig. S3.** The expression levels of IL-10, IL-4, IL-1β, and TNF-α in cell suspension of RAW246.7 with dexamethasone in different concentrations.

**Fig. S4**. Cell viability showing cytotoxicity of RAW246.7 with dexamethasone in different concentrations.

**Fig. S5.** Unprocessed western blot image corresponding to **Fig. 2a**.

**Fig. S6**. Plasmid map of pBudCE4.1-Hp-EGFP&IL-10-mCherry.

**Fig. S7.** Mean fluorescence intensity quantification of Hp and IL-10 was measured in cells treated with Lipo 2000@Vector, Lipo 2000@HI, or M2‑exo@HI.

**Fig. S8.** Loading efficiency, plasmid integrity, and expression activity under different voltage. (a) The loading efficiency of M2-exo@HI at different voltage parameters. (b) DNA agarose gel electrophoresis showing the plasmid integrity at different voltages. (c) Fluorescence microscope showing the expression of Hp (green) and IL-10 (red) in BV2 cells at different groups.

**Fig. S9.** Stability evaluation of M2-exo@HI in PBS at 4 ℃ by monitoring zeta potentials over time.

**Fig. S10.** In vitro biocompatibility evaluation. (a) Live/dead staining fluorescence images of EC and BV_2_ cells treated with different concentrations of M2-exo@HI. (b) Hemolysis and (c) coagulation of M2exo@HI at different concentrations.

**Fig. S11.** Gating strategies for flow cytometric analysis of (a) M1 microglia (eFlour 660^-^/CD86^+^) and (b) M2 microglia (eFlour 660^-^/CD206^+^) in LPS induced microglia polarization.

**Fig. S12.** Representative flow cytometry plot of the M2-type microglia (labeled with CD206^+^) and M1-type microglia (CD86^+^) among BV2 cells with or without LPS treatment.

**Fig. S13.** (a) Fluorescence images and (b) quantitative analysis of ICG and M2-exo@ICG phagocytosed by M1 microglia at different time points.

**Fig. S14.** The expression levels of Hp (a) and IL-10 (b) in cell suspension from M1-type microglia following transfection with Lipo@HI or M2-exo@HI after 24 hours.

**Fig. S15.** Unprocessed western blot image corresponding to IL -10 in **Fig. 3j**.

**Fig. S16.** (a) Fluorescence images and (b) quantitative analysis of apoptotic RBC (labeled with Annexin^+^) with or without t-BHP treatment.

**Fig. S17.** Representative flow cytometry plot of the RBC Labeled with or without DiO.

**Fig. S18.** Schematic of microglia-mediated neuroprotection using flow cytometry.

**Fig. S19.** Preferential uptake of M2-exo@RhB by microglia, astrocyte, and neurons in the hemorrhagic brain. (a) Representative flow cytometry histograms showing RhB fluorescence in brain-resident microglia (Iba-1^+^), astrocytes (GFAP^+^), and neurons (NeuN^+^) after administration 24 h of M2-exo@RhB, M2-exo@RhB+CD206, and M2-exo@RhB+RGD. (b) Quantification of RhB^+^ cells (%) in different groups.

**Fig. S20.** In vivo brain accumulation of targeted M2‑exo formulations in ICH mice. (a) In vivo near-infrared fluorescence imaging showing M2-exo@ICG, M2-exo@ICG+CD206, and M2-exo@ICG+RGD in mouse brains at various time points post-injection. (b) Average radiation efficiency of ICG in different treatment groups.

**Fig. S21.** In vivo two‑photon imaging of M2‑exo@RhB in the peri‑hematomal cortex of ICH mice.

**Fig. S22.** Immunofluorescence staining showing co-localization of Hp/IL-10 with Neuron.

**Fig. S23.** In vivo therapeutic effects of different doses of M2-exo@HI in hemorrhagic stroke. (a) Digital photos showing cerebral hematoma of ICH mice in different groups. (b) Quantitative measurements of hemoglobin concentration in different groups.

**Fig. S24.** The inflammatory cytokine levels of IL-10, TGF-β, IL-1β, and TNF-𝛼 in different groups.

**Fig. S25.** The average optical density of HE, Nissl, and TUNEL staining.

**Fig. S26.** Digital photos showing Evans blue extravasation. (a) and quantitative measurements (b) of Evans blue content in different groups.

**Fig. S27.** Long-term neurological functional outcome of ICH mice with M2-exo@HI treatment. (a) Schematic of the balance beam test, and the mNSS evaluation. (b) Photograph of the rotating cylinder test, and the latency to fall in the test. (c) Photograph of the cylinder test, and the forelimb asymmetry rate in the test. (d) Photograph of the adhesive test, and the time to remove the tape in the test.

**Fig. S28.** (a) Schematic of the Morris water maze test, and the motion path of mice in the test at day 28 after ICH. (b) Number of platform crossings and (c) escape latency of Morris water maze test.

**Fig. S29.** Principal component analysis (PCA) revealed distinct clustering patterns among sham, ICH, and M2-exo@HI groups.

**Fig. S30.** (a) Representative WB iamges of Cd163, Ikbke, Cxcr4 and Igf2 in peri‑hematomal brain tissues from Sham, ICH, M2‑exo@HI and M2‑exo@HI+PMA groups. (b) Heat map summarizing the relative protein expression levels normalized to β‑actin.

**Fig. S31.** (a) Representative flow cytometry plot of the CD4^+^ T cell in different groups. (b) Quantitative measurements the percentages of CD4^+^ T cells in different groups.

**Fig. S32.** HE staining of heart, liver, spleen, lung and kidney harvested from mice in different groups.

**Fig. S33.** Analysis of blood count and liver and kidney function of mice treated with different groups. 1, 2, and 3 represent PBS for 3 days, M2-exo@HI for 3 days, and M2-exo@HI for 28 days, respectively.

**Fig. S34.** The long-term safety and persistence of plasmid expression in mice. (a) qPCR analysis of HI plasmid clearance in mouse tissues at different time points post-injection. (b) Body temperature of mice at 30 min after the first, second, and third injections of M2-exo@HI. (c) TNF-αand IL-8 levels in serum at 15 min after the third dose with M2-exo@HI.

**Fig. S35.** The long-term clearance of M2-exo@ICG in different tissues. (a) Ex vivo fluorescence imaging of M2-exo@ICG in mouse tissues at different time points post-injection. (b) The average radiation efficiency of M2-exo@ICG in mouse tissues based on fluorescence intensity.


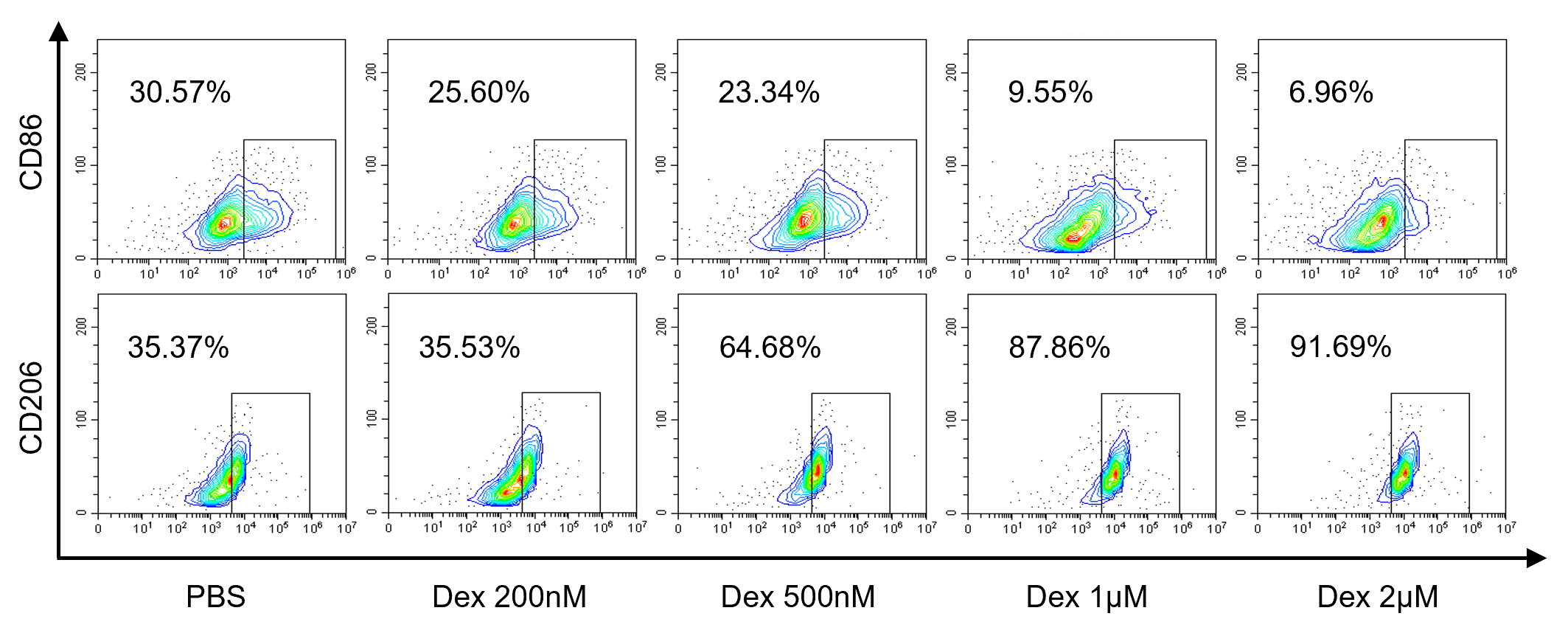


**Fig. S1.** Representative flow cytometry plot of the M2-type macrophage (labeled with CD206^+^) and M1-type macrophage (labeled with CD86^+^) among RAW264.7 with dexamethasone in different concentrations.


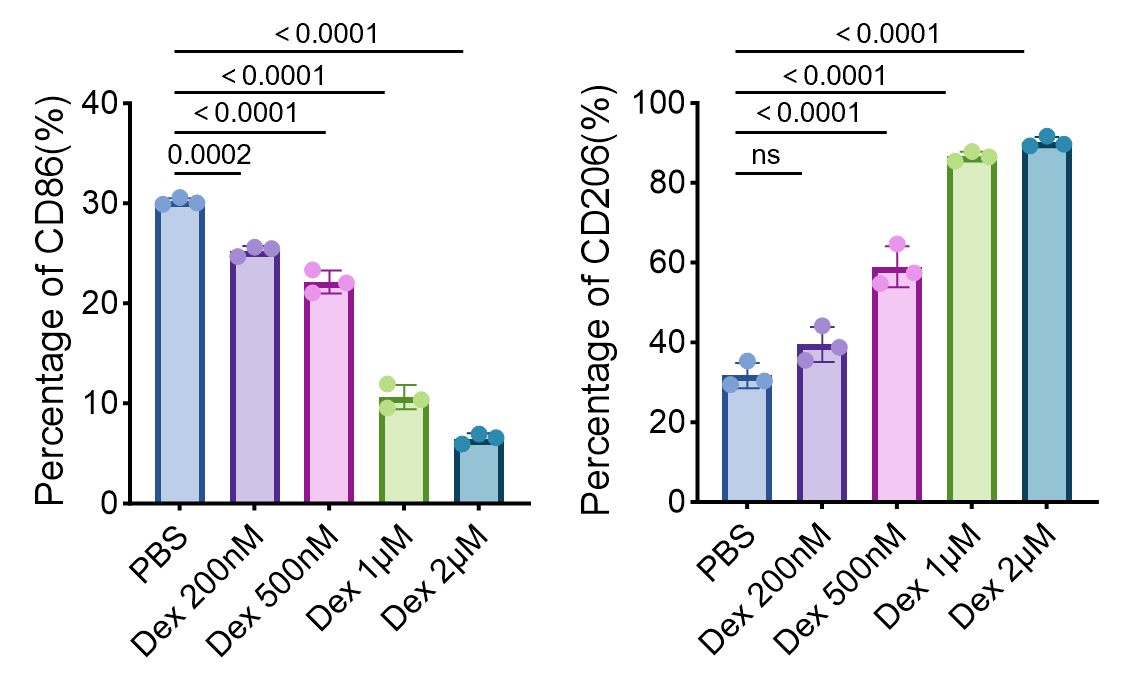


**Fig. S2.** Percentages of CD86^+^ and CD206^+^ cells in different groups (n = 3). Data are presented as mean ± SD. Statistical significance was tested by one-way ANOVA with Tukey’s multiple comparisons test.


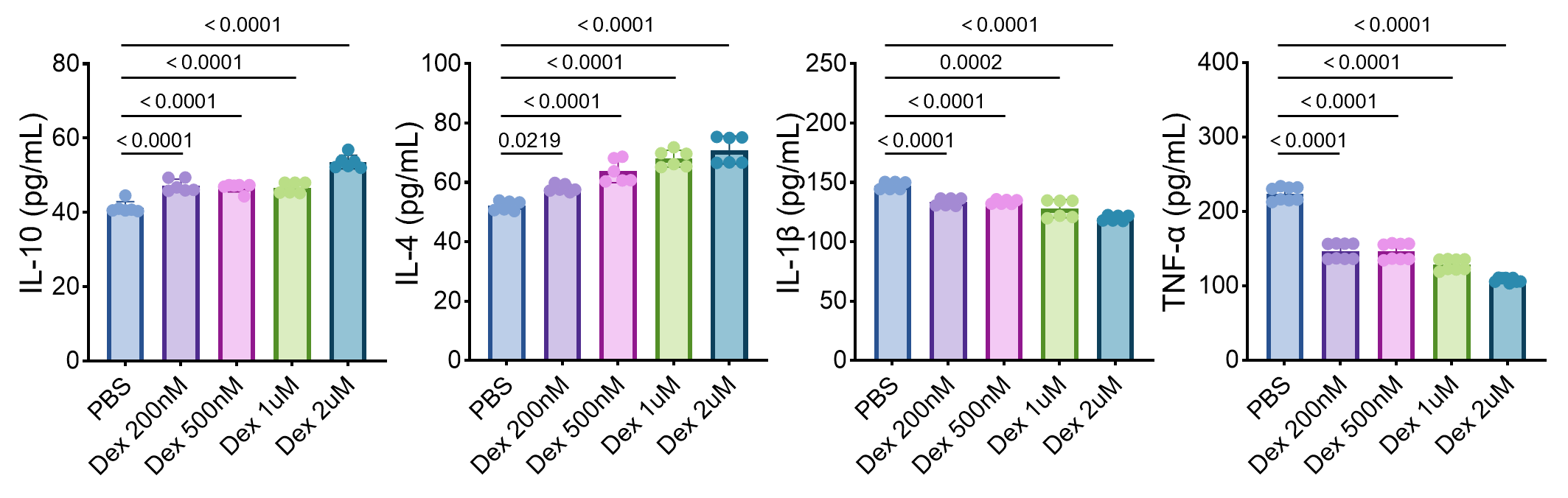


**Fig. S3.** The expression levels of IL-10, IL-4, IL-1β, and TNF-α in cell suspension of RAW246.7 with dexamethasone in different concentrations (n = 6). Data are presented as mean ± SD. Statistical significance was tested by one-way ANOVA with Tukey’s multiple comparisons test.


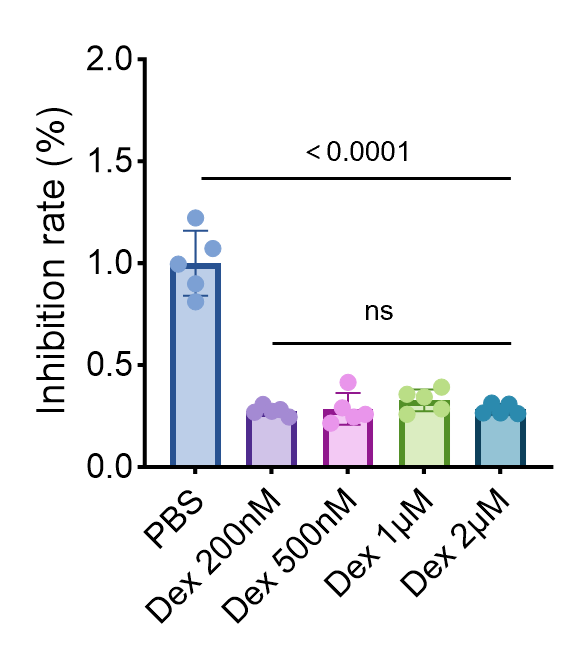


**Fig. S4**. Cell viability showing cytotoxicity of RAW246.7 with dexamethasone in different concentrations (n = 6). Data are presented as mean ± SD. Statistical significance was tested by one-way ANOVA with Tukey’s multiple comparisons test.


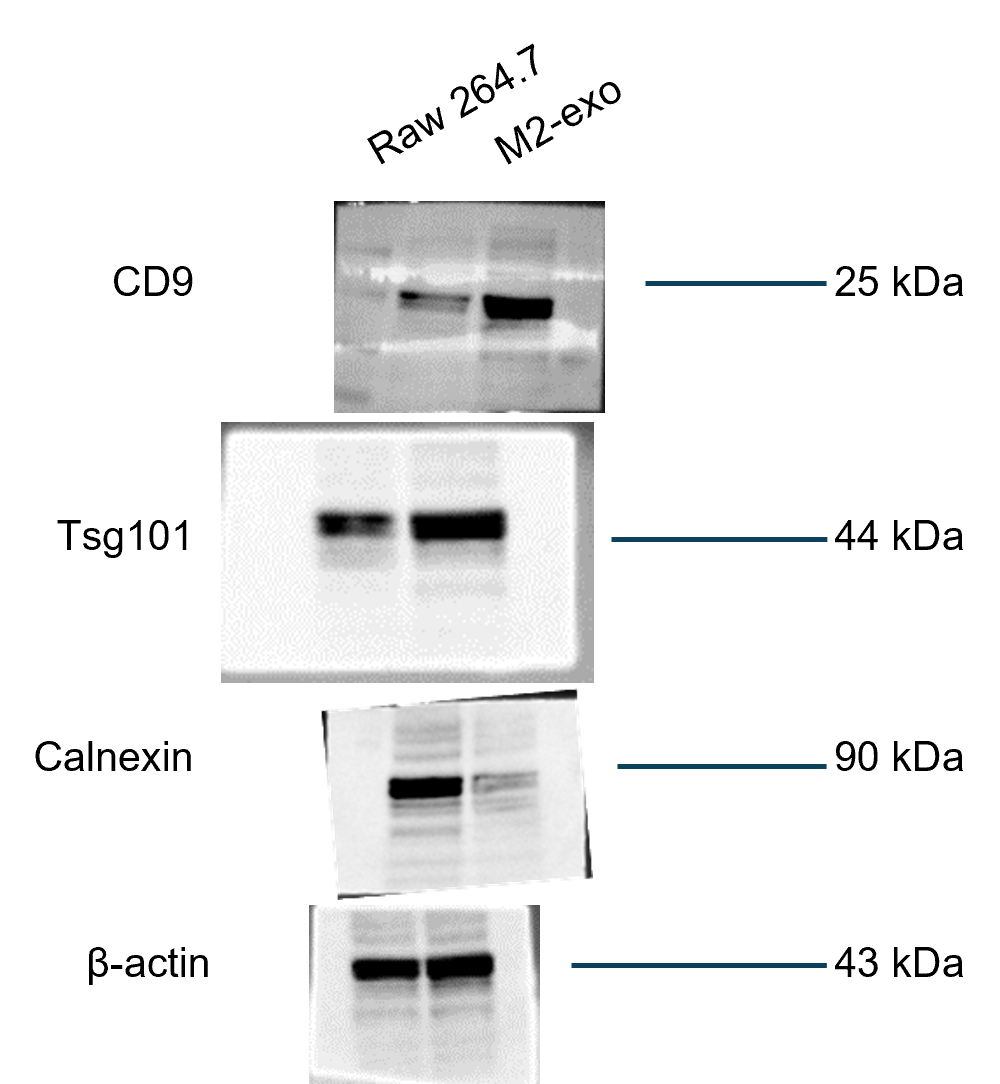


**Fig. S5.** Unprocessed western blot image corresponding to Fig. 2a.


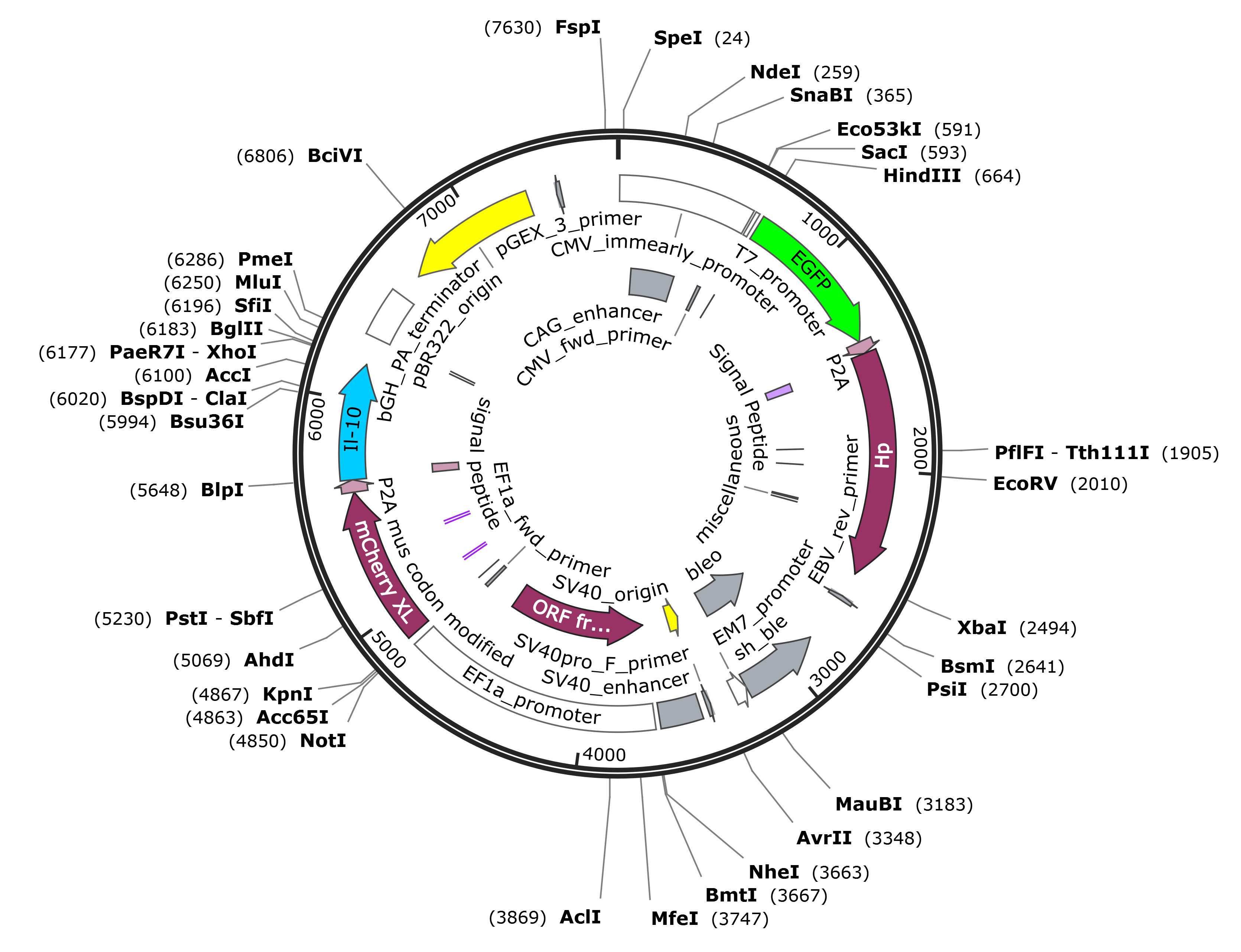


**Fig. S6**. Plasmid map of pBudCE4.1-Hp-EGFP&IL-10-mCherry.


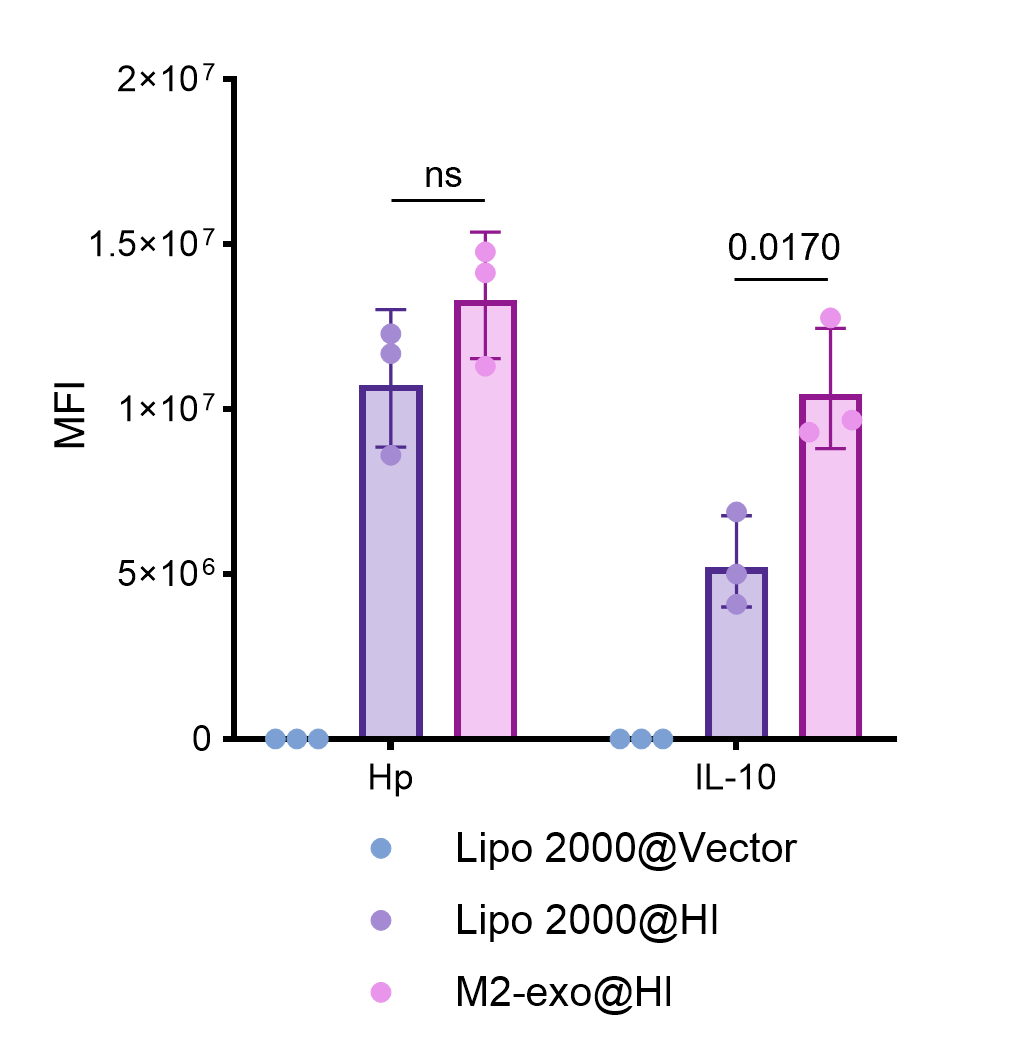


**Fig.S7.** Mean fluorescence intensity quantification of Hp and IL-10 was measured in cells treated with Lipo 2000@Vector, Lipo 2000@HI, or M2‑exo@HI (n = 3). Data are presented as mean ± SD. Statistical significance was tested by one-way ANOVA with Tukey’s multiple comparisons test.


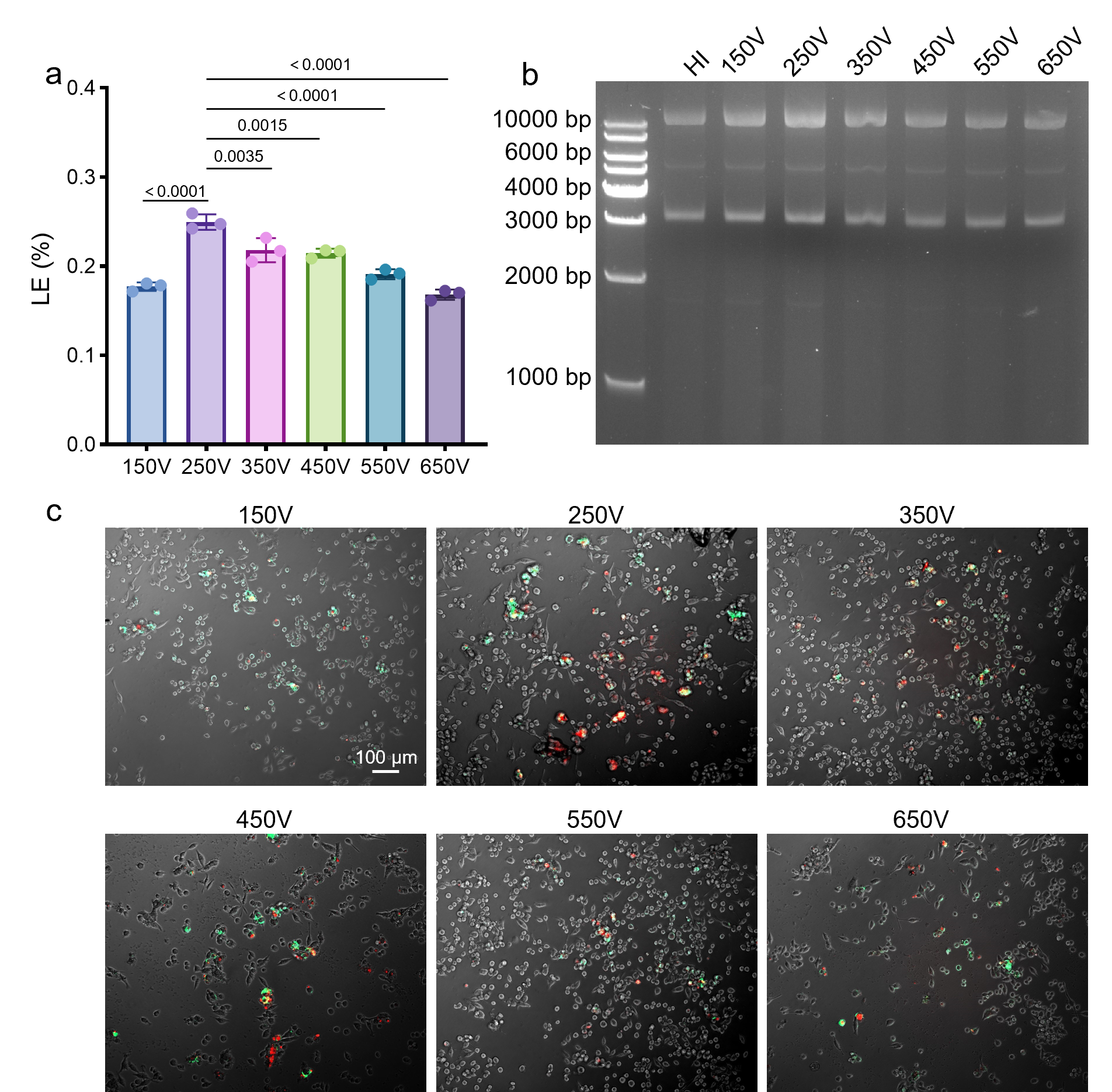


**Fig. S8.** Loading efficiency, plasmid integrity, and expression activity under different voltage. (a) The loading efficiency of M2-exo@HI at different voltage parameters (n = 3). (b) DNA agarose gel electrophoresis showing the plasmid integrity at different voltages. (c) Fluorescence microscope showing the expression of Hp (green) and IL-10 (red) in BV2 cells at different groups. Data are presented as mean ± SD. Statistical significance was tested by one-way ANOVA with Tukey’s multiple comparisons test.


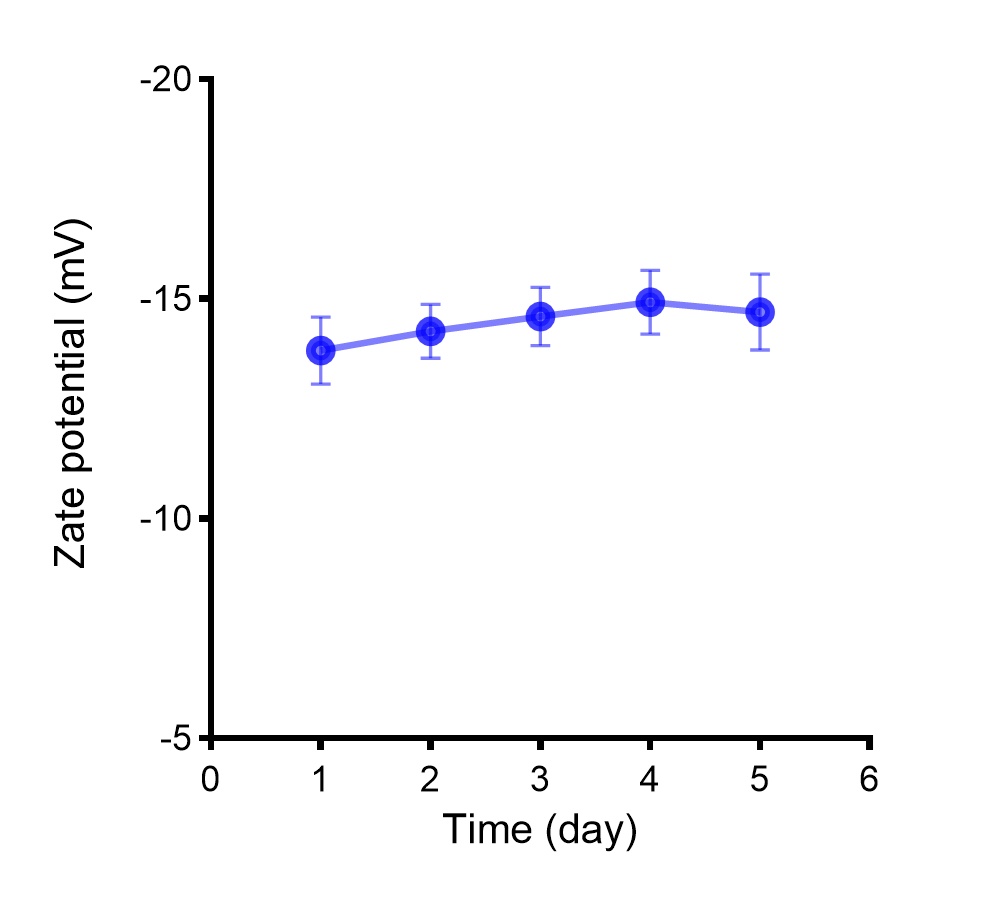


**Fig. S9.** Stability evaluation of M2-exo@HI in PBS at 4 ℃ by monitoring zeta potentials over time (n = 3).


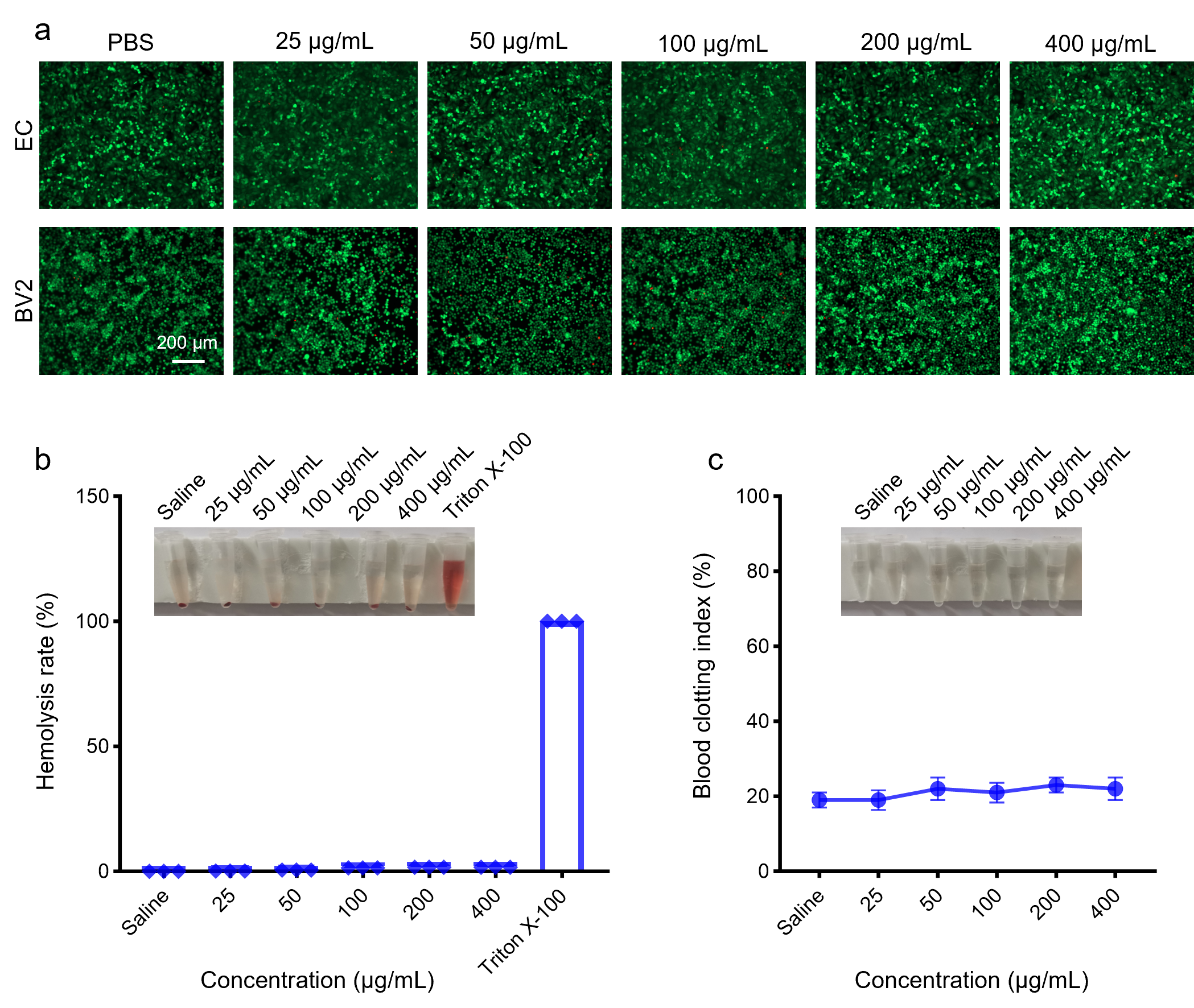


**Fig. S10.** In vitro biocompatibility evaluation. (a) Live/dead staining fluorescence images of EC and BV2 cells treated with different concentrations of M2-exo@HI. (b) Hemolysis and (c) coagulation of M2exo@HI at different concentrations (n = 3).


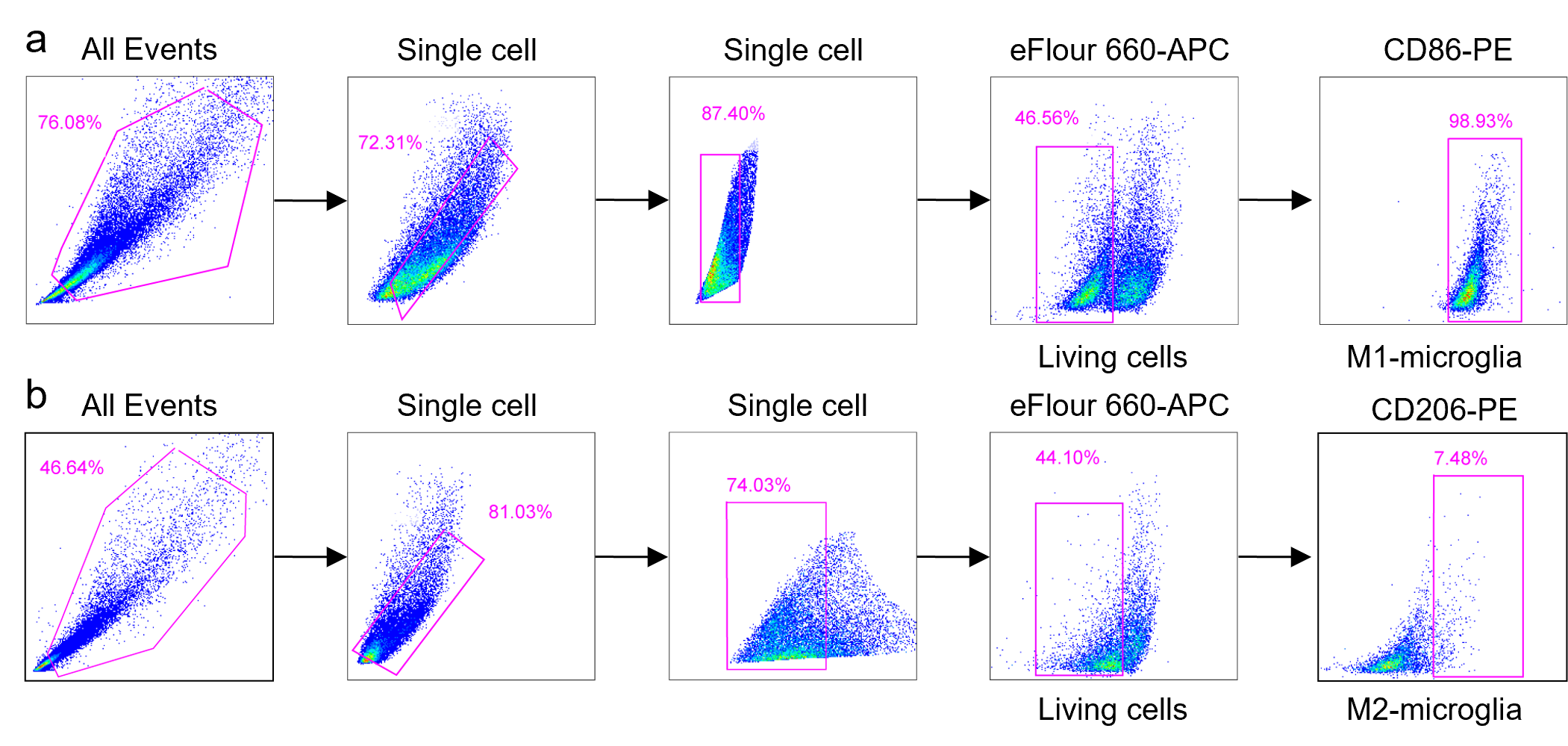


**Fig. S11.** Gating strategies for flow cytometric analysis of (a) M1 microglia (eFlour 660^-^/CD86^+^) and (b) M2 microglia (eFlour 660^-^/CD206^+^) in LPS induced microglia polarization.

**
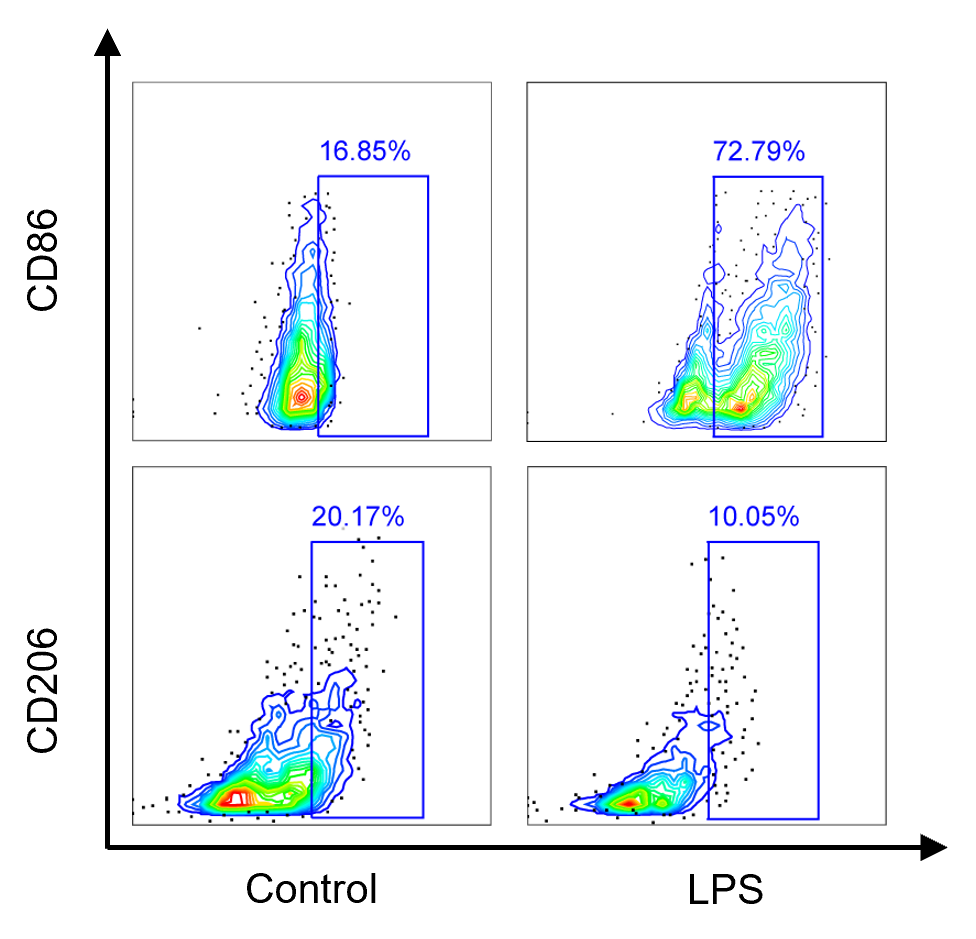
**

**Fig. S12.** Representative flow cytometry plot of the M2-type microglia (labeled with CD206^+^) and M1-type microglia (CD86^+^) among BV2 cells with or without LPS treatment.


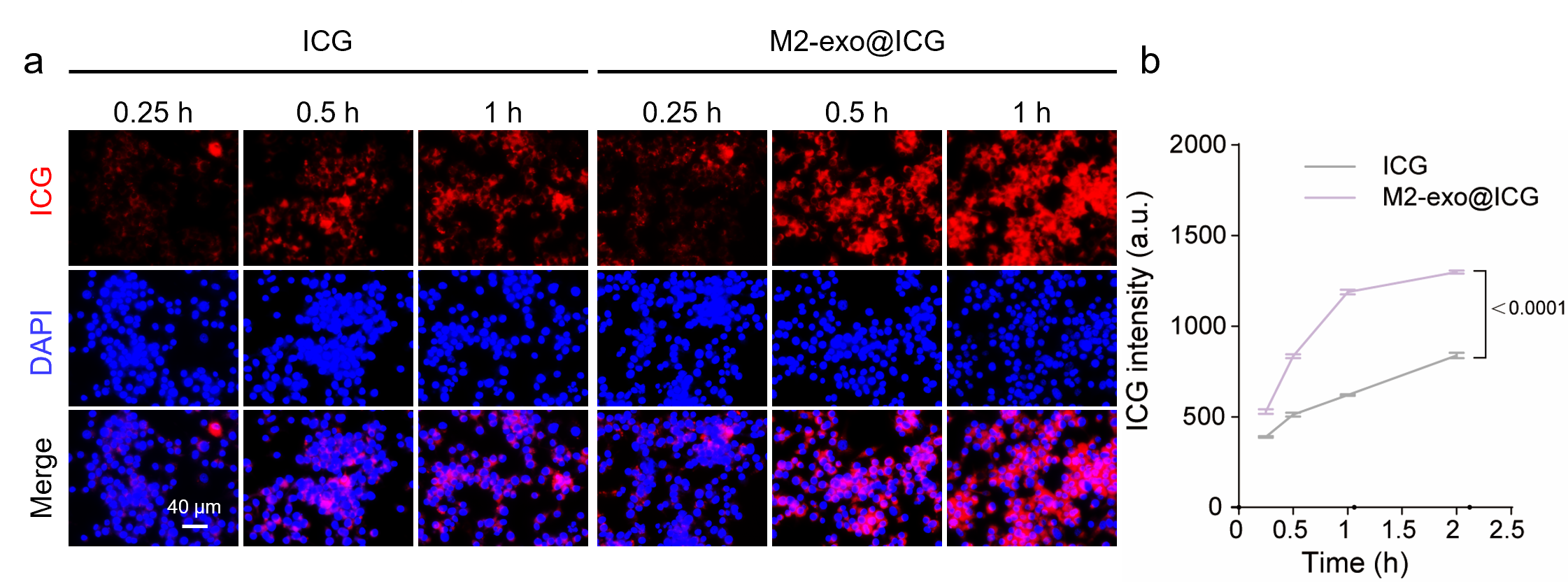


**Fig. S13.** (a) Fluorescence images and (b) quantitative analysis of ICG and M2-exo@ICG phagocytosed by M1 microglia at different time points (n = 3). Data are presented as mean ± SD. Statistical significance was tested by two-way ANOVA with Bonferroni’s multiple comparisons test.


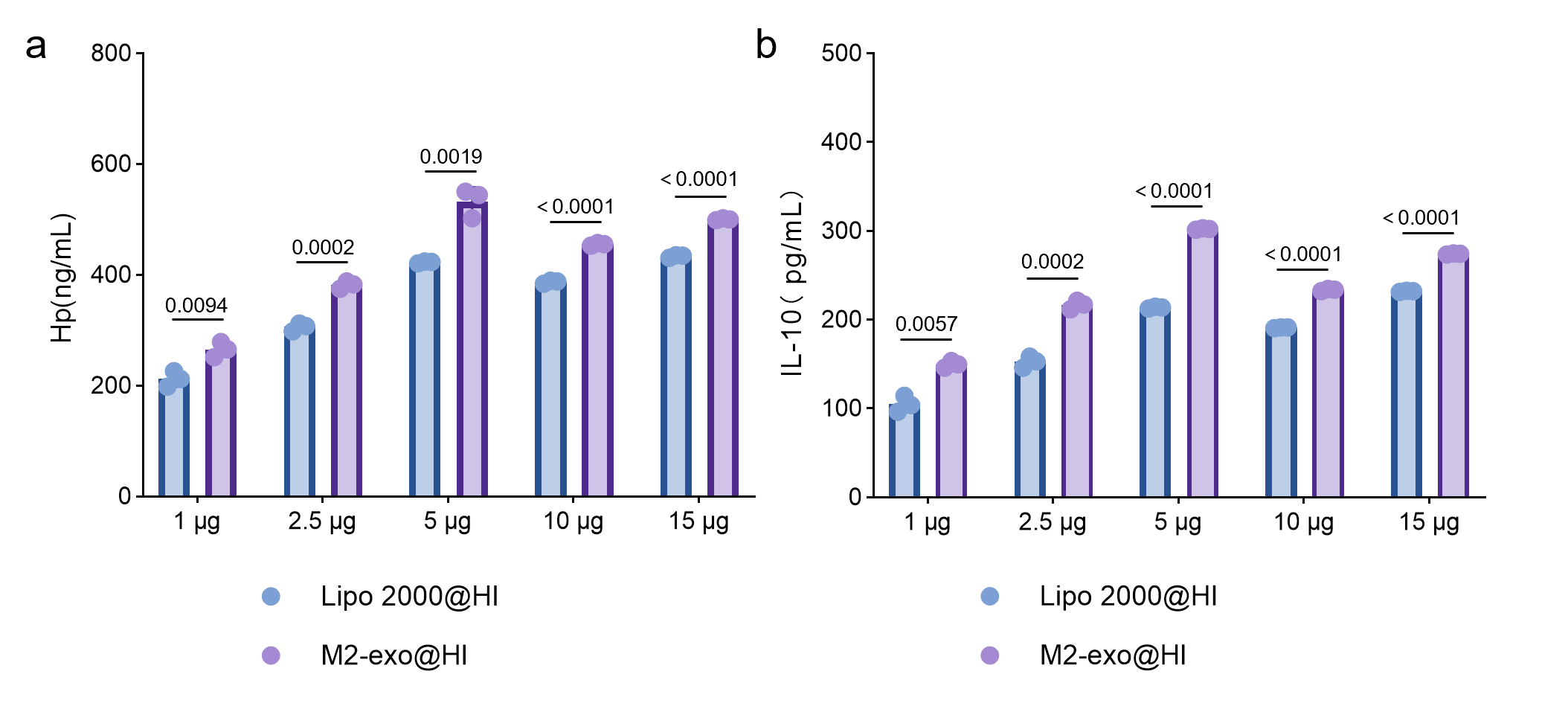


**Fig. S14.** The expression levels of (a) Hp and (b) IL-10 in cell suspension from M1-type microglia following transfection with Lipo@HI or M2-exo@HI after 24 hours (n = 3). Data are presented as mean ± SD. Statistical significance was tested by unpaired Student’s *t*-test.


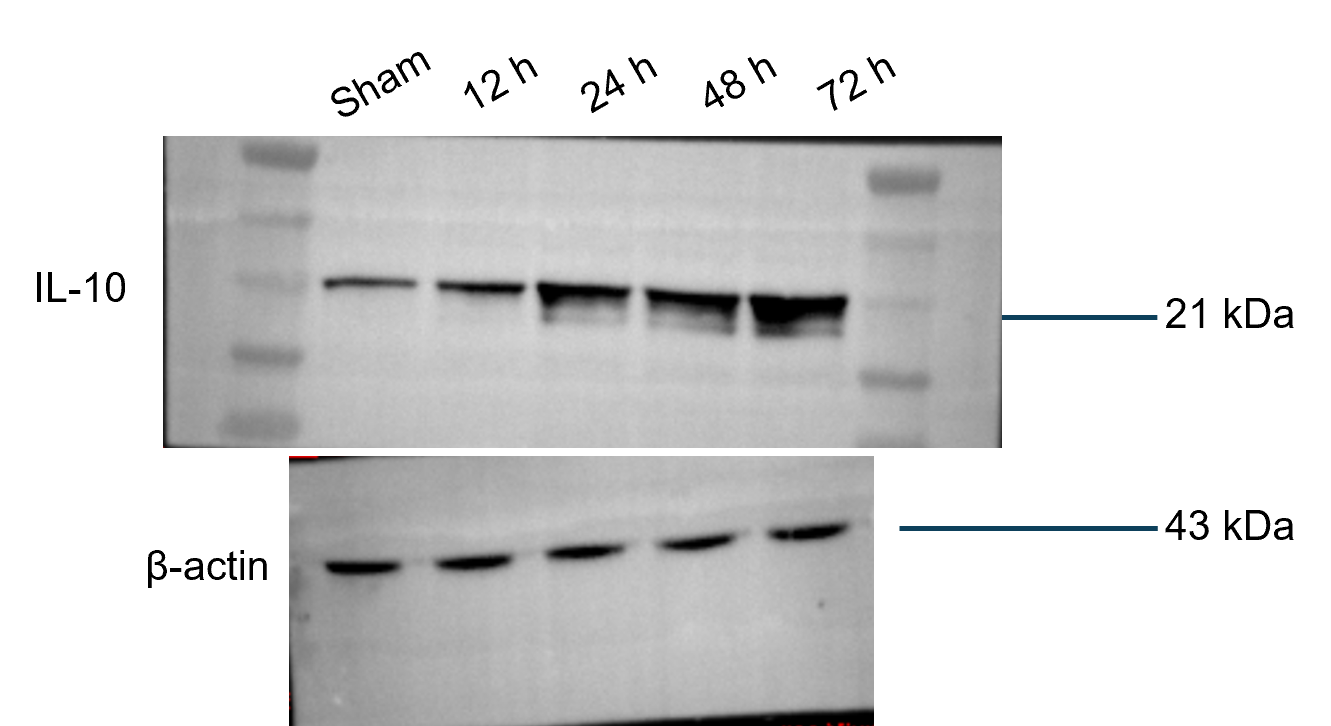


**Fig. S15.** Unprocessed western blot image corresponding to IL -10 in **Fig. 3j**.


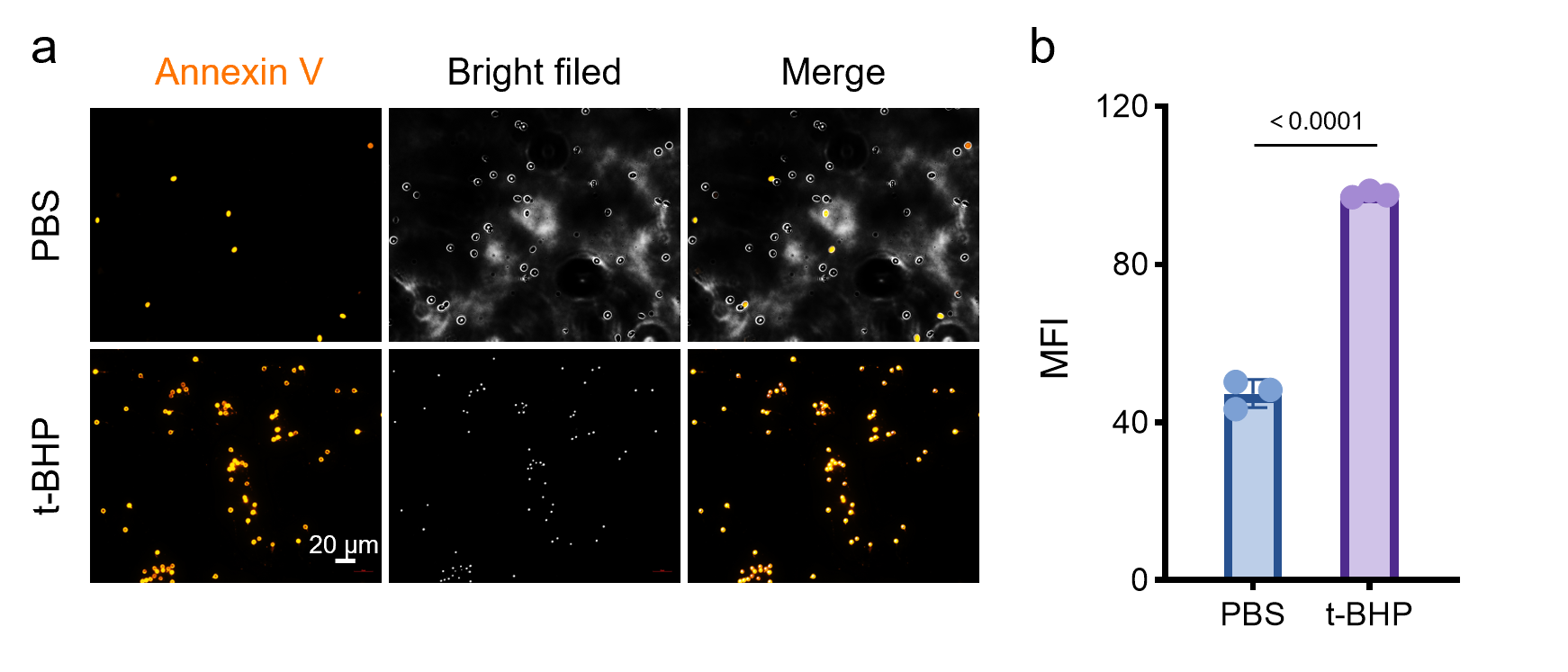


**Fig. S16**. (a) Fluorescence images and (b) quantitative analysis of apoptotic RBC (labeled with Annexin^+^) with or without t-BHP treatment (n = 3). Data are presented as mean ± SD. Statistical significance was tested by unpaired Student’s *t*-test.


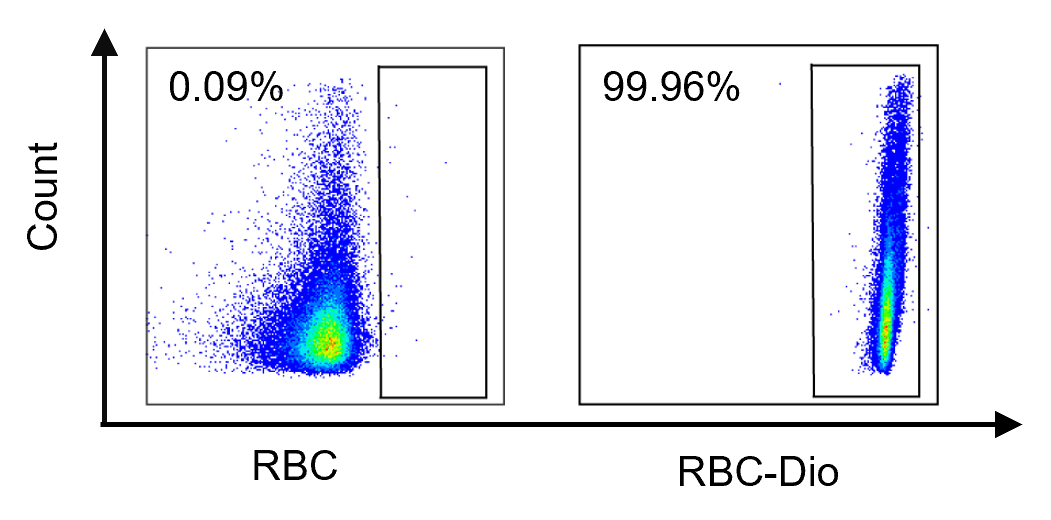


**Fig. S17**. Representative flow cytometry plot of the RBC Labeled with or without DiO.


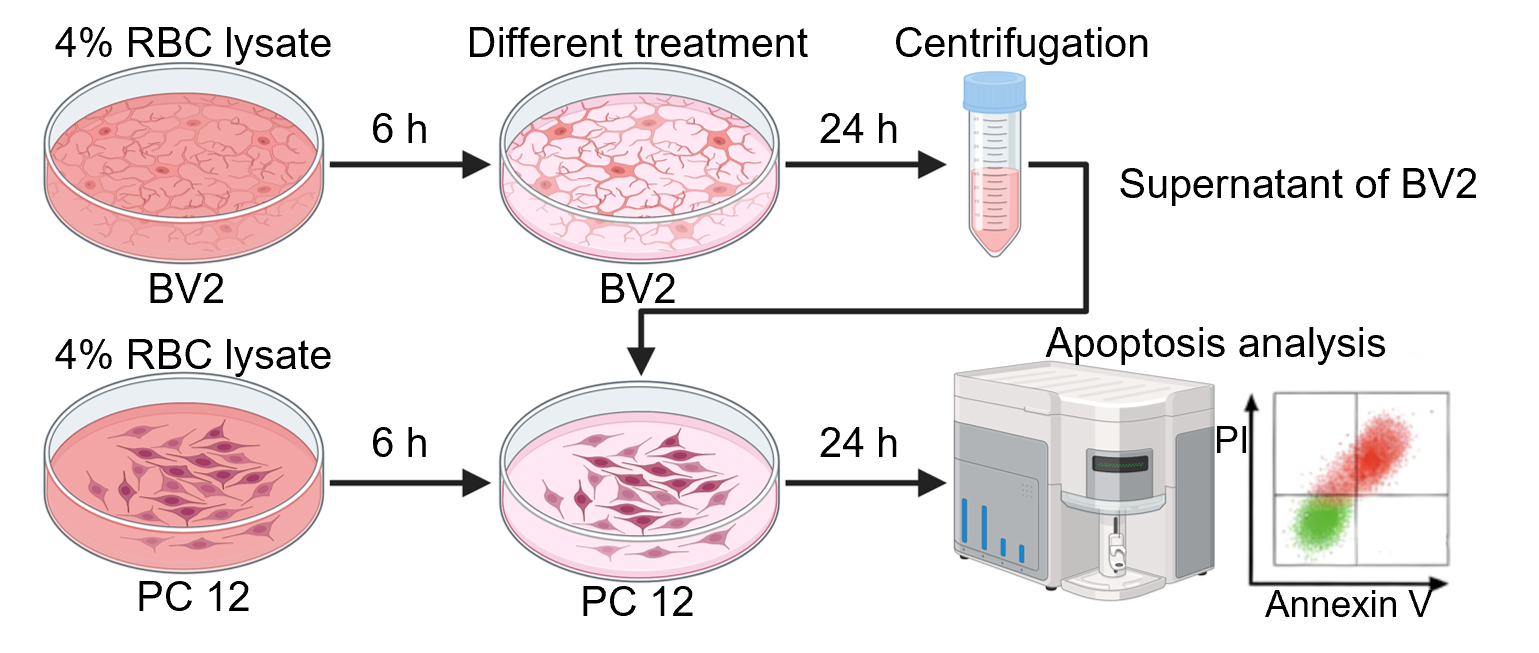


**Fig. S18.** Schematic of microglia-mediated neuroprotection using flow cytometry.

**
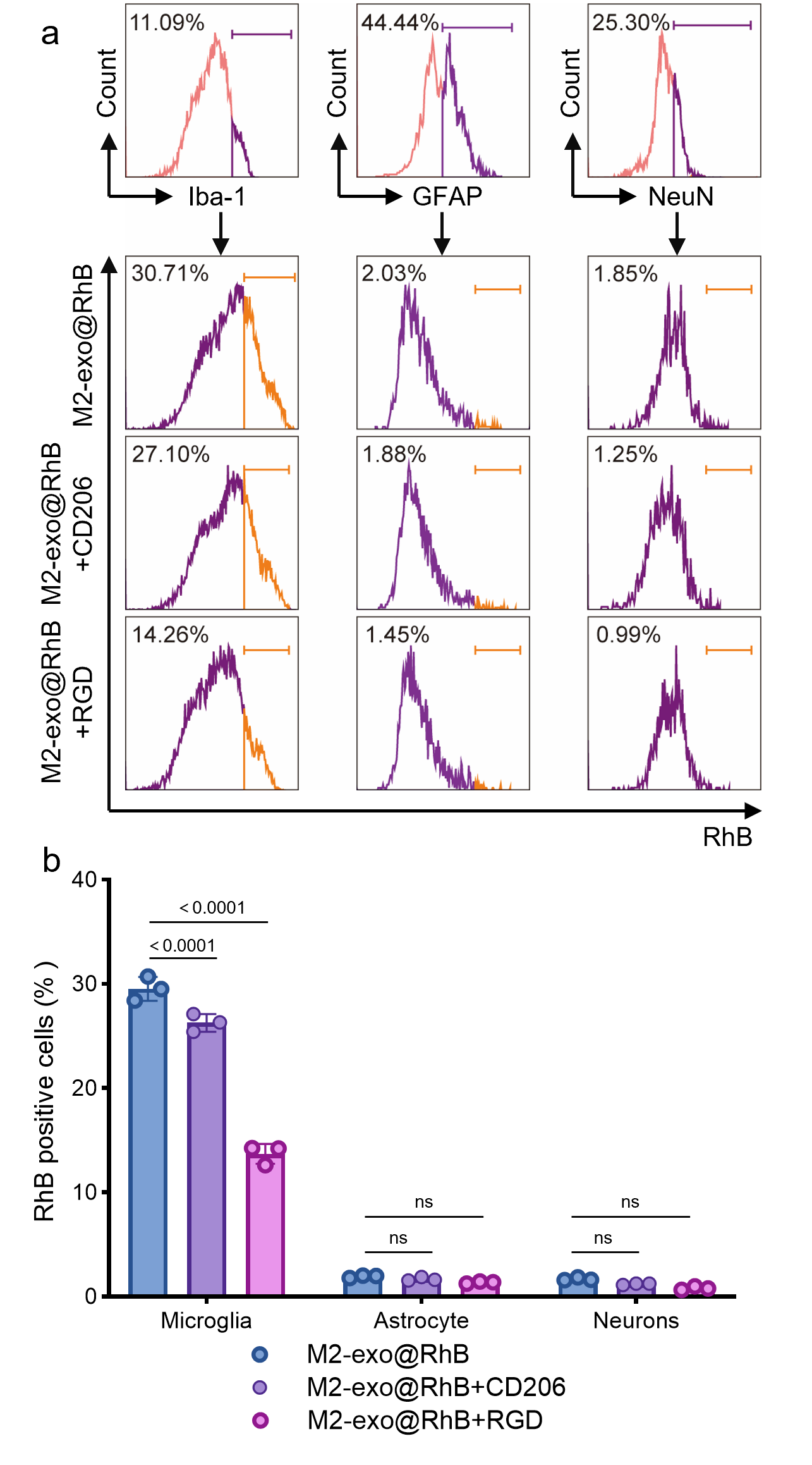
**

**Fig. S19.** Preferential uptake of M2-exo@RhB by microglia, astrocyte, and neurons in the hemorrhagic brain. (a) Representative flow cytometry histograms showing RhB fluorescence in brain-resident microglia (Iba-1^+^), astrocytes (GFAP^+^), and neurons (NeuN^+^) after administration 24 h of M2-exo@RhB, M2-exo@RhB+CD206, and M2-exo@RhB+RGD. (b) Quantification of RhB+ cells (%) in different groups (n = 3). Data are presented as mean ± SD. Statistical significance was tested by one-way ANOVA with Tukey’s multiple comparisons test.


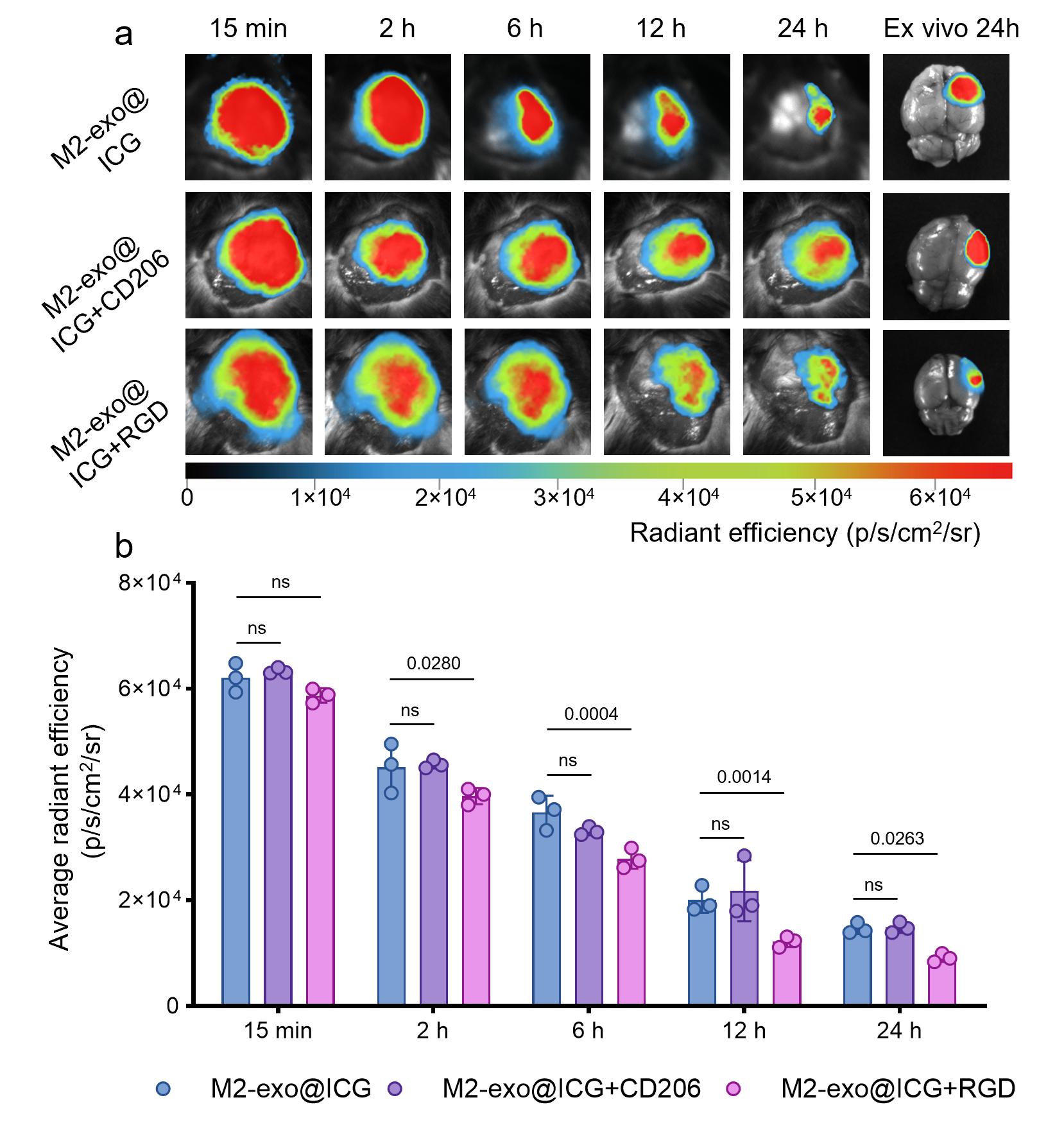


**Fig. S20.** In vivo brain accumulation of targeted M2‑exo formulations in ICH mice. (a) In vivo near-infrared fluorescence imaging showing M2-exo@ICG, M2-exo@ICG+CD206, and M2-exo@ICG+RGD in mouse brains at various time points post-injection. (b) Average radiation efficiency of ICG in different treatment groups (n = 3). Data are presented as mean ± SD. Statistical significance was tested by one-way ANOVA with Tukey’s multiple comparisons test.


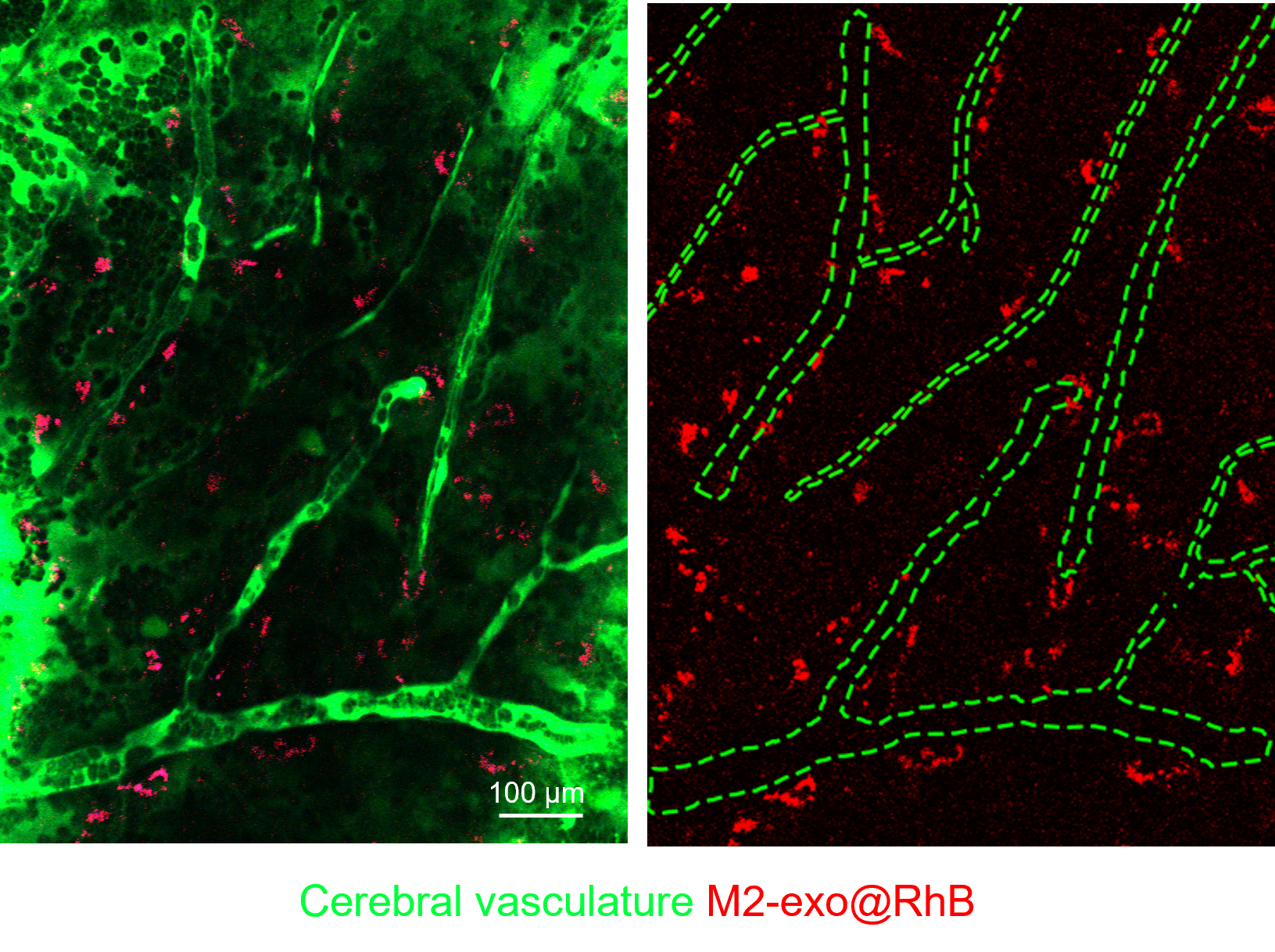


**Fig. S21.** In vivo two‑photon imaging of M2‑exo@RhB in the peri‑hematomal cortex of ICH mice. Cerebral vasculature (Evans blue, green), M2‑exo (RhB, red).

**
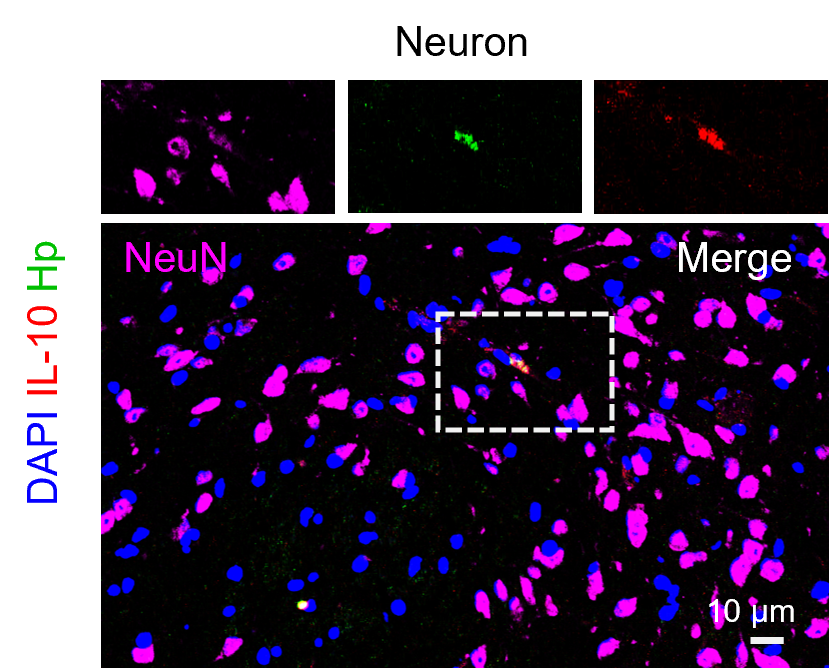
**

**Fig. S22.** Immunofluorescence staining showing co-localization of Hp/IL-10 with Neuron.


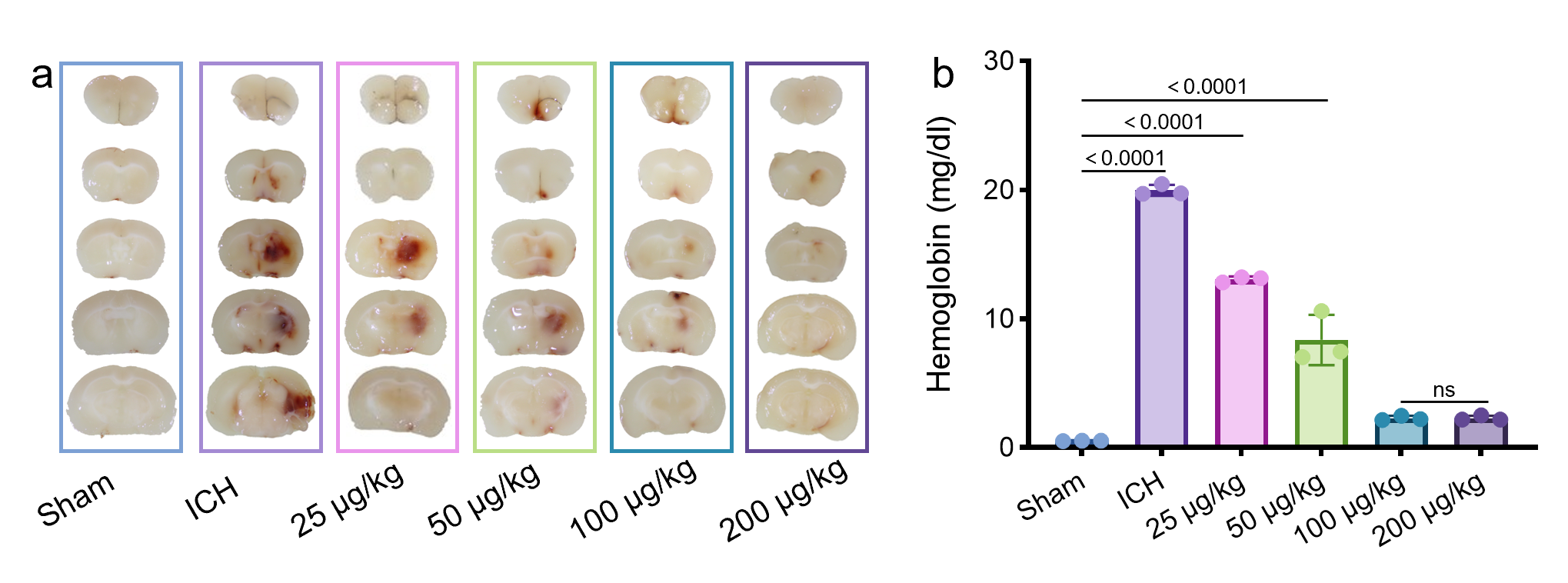


**Fig. S23.** In vivo therapeutic effects of different doses of M2-exo@HI in hemorrhagic stroke. (a) Digital photos showing cerebral hematoma of ICH mice in different groups. (b) Quantitative measurements of hemoglobin concentration in different groups (n = 3). Data are presented as mean ± SD. Statistical significance was tested by one-way ANOVA with Tukey’s multiple comparisons test.

**
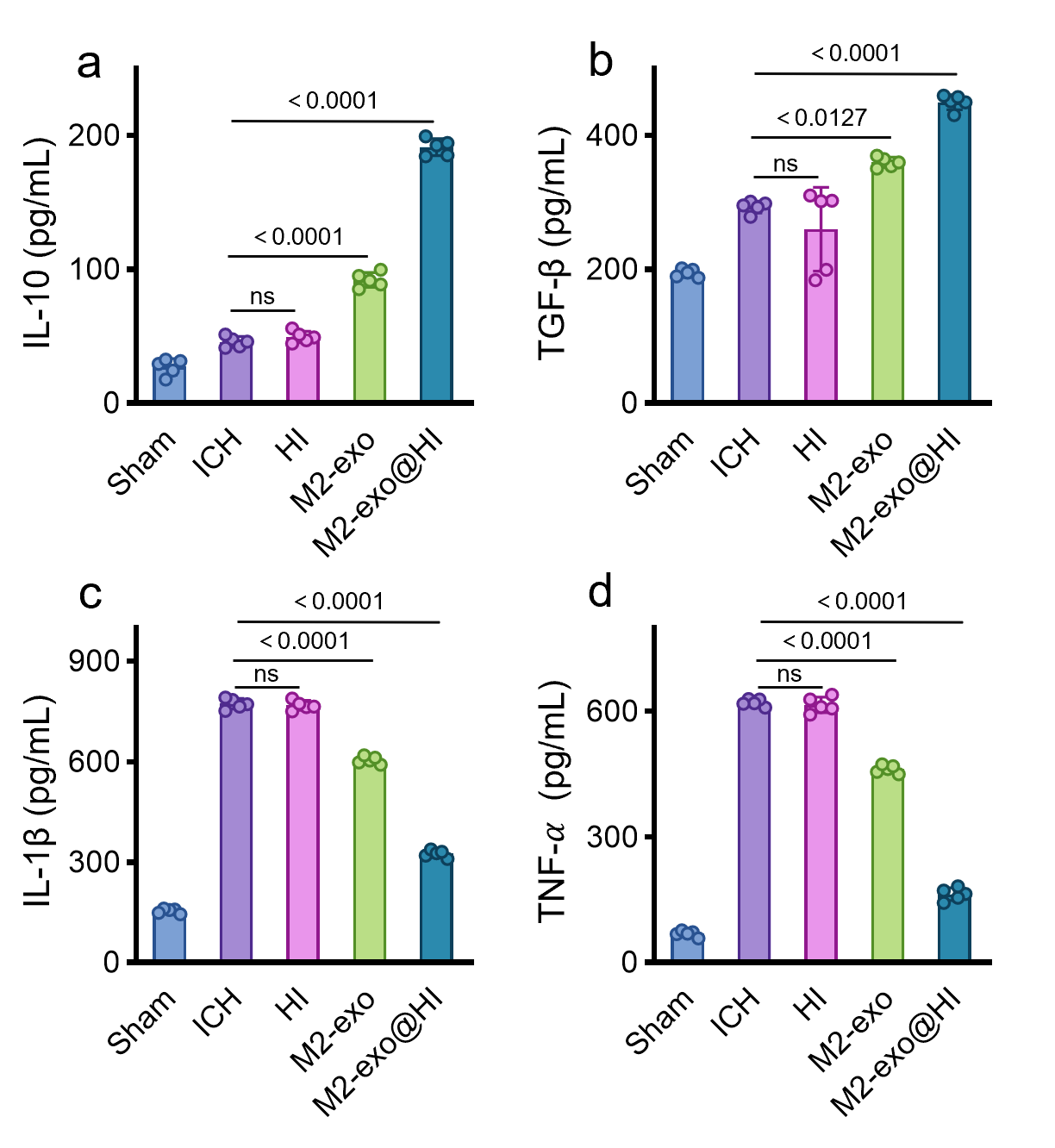
**

**Fig. S24.** The inflammatory cytokine levels of IL-10, TGF-β, IL-1β, and TNF-𝛼 in different groups (n = 5). Data are presented as mean ± SD. Statistical significance was tested by one-way ANOVA with Tukey’s multiple comparisons test.


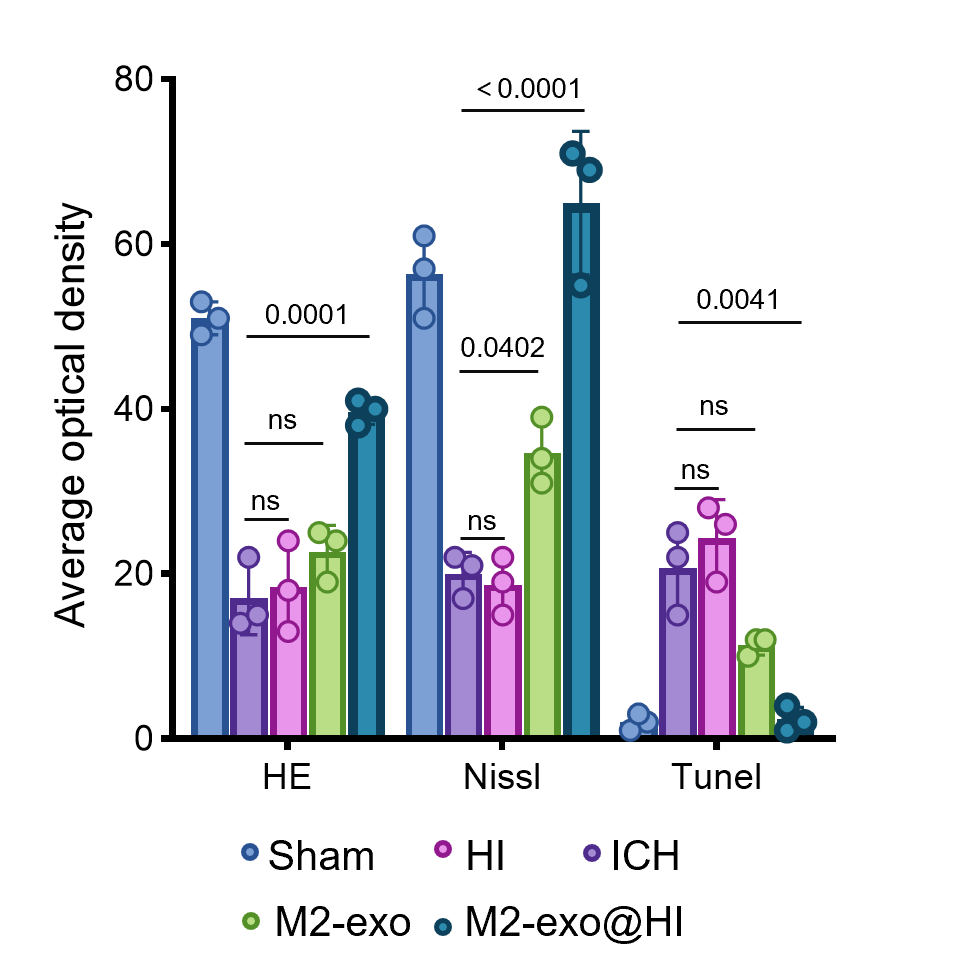


**Fig. S25.** The average optical density of HE, Nissl, and TUNEL staining (n = 3). Data are presented as mean ± SD. Statistical significance was tested by one-way ANOVA with Tukey’s multiple comparisons test.


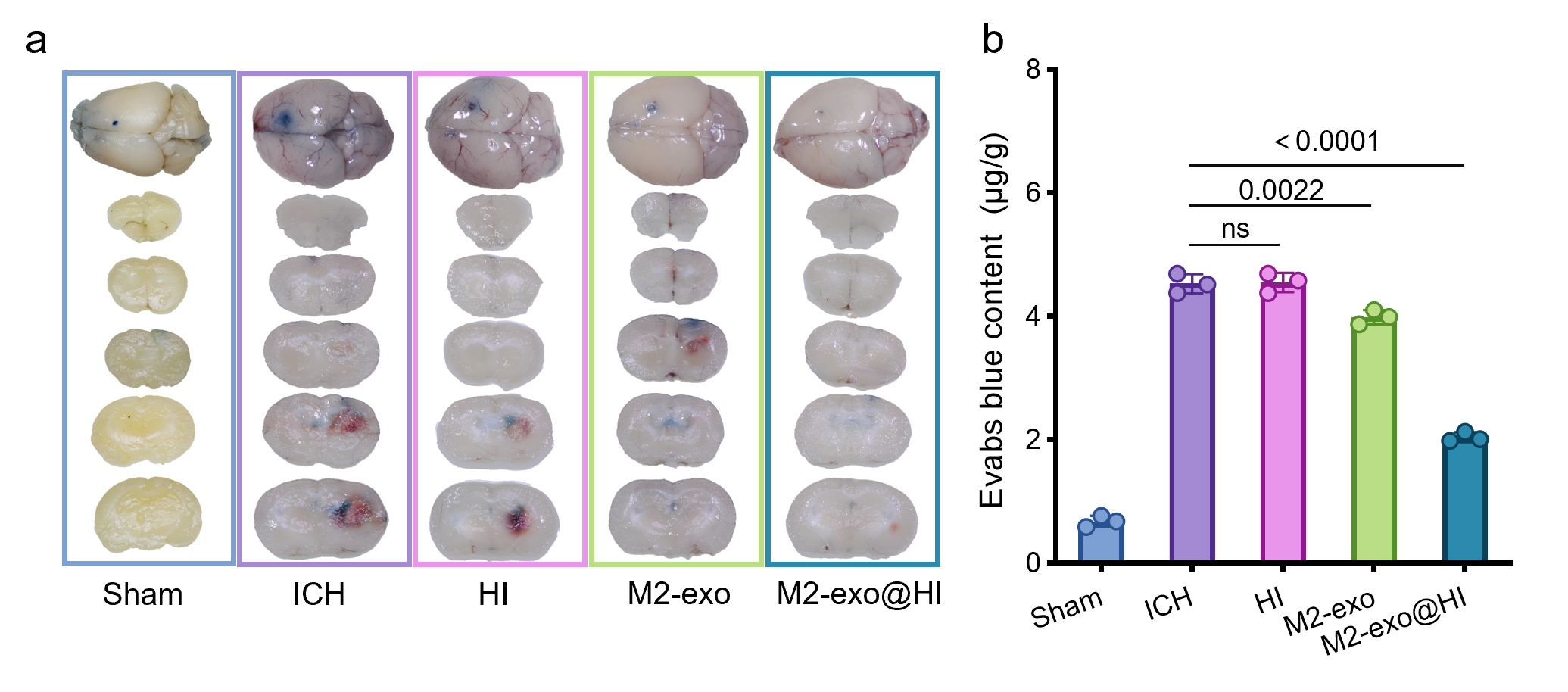


**Fig. S26.** Digital photos showing Evans blue extravasation. (a) and quantitative measurements (b) of Evans blue content in different groups (n = 3). Data are presented as mean ± SD. Statistical significance was tested by one-way ANOVA with Tukey’s multiple comparisons test.


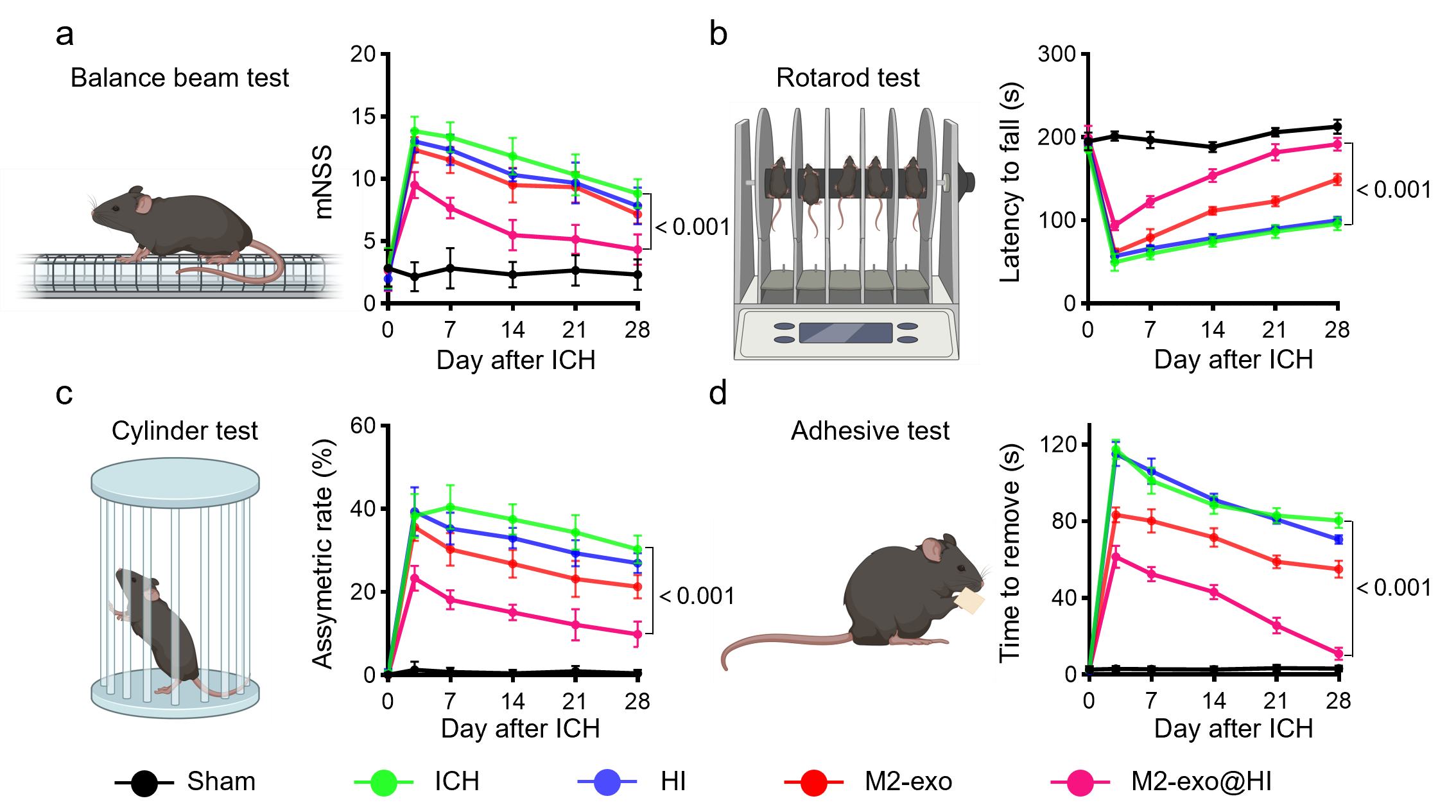


**Fig. S27.** Long-term neurological functional outcome of ICH mice with M2-exo@HI treatment. (a) Schematic of the balance beam test, and the mNSS evaluation (n = 6). (b) Photograph of the rotating cylinder test, and the latency to fall in the test (n = 6). (c) Photograph of the cylinder test, and the forelimb asymmetry rate in the test n = 6). (d) Photograph of the adhesive test, and the time to remove the tape in the test (n = 6). Data are presented as mean ± SD. Statistical significance was tested by two-way ANOVA with Bonferroni’s multiple comparisons test.


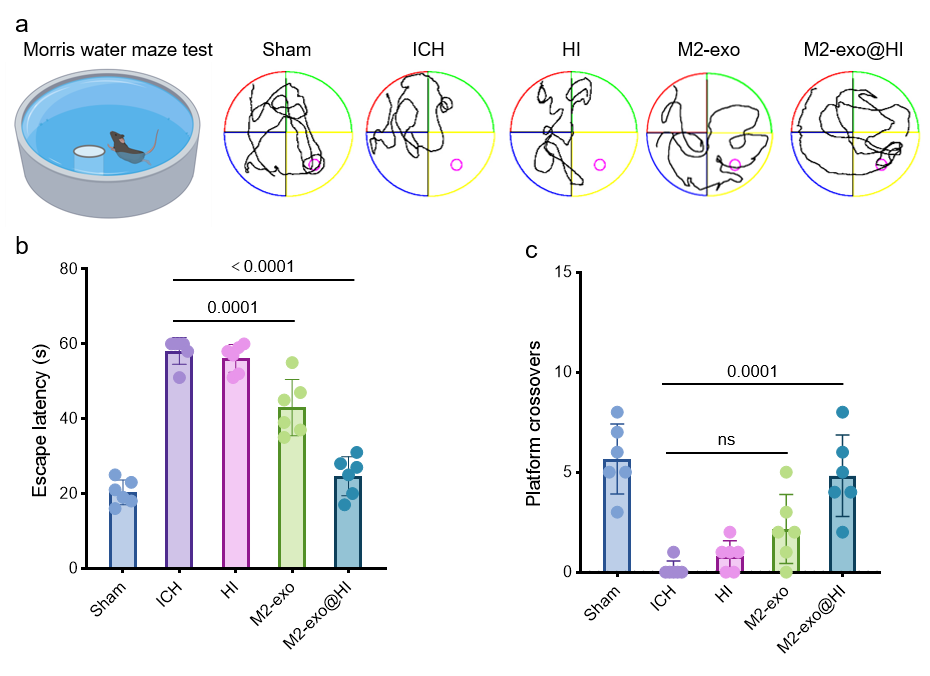


**Fig. S28.** (a) Schematic of the Morris water maze test, and the motion path of mice in the test at day 28 after ICH. (b) Number of platform crossings and (c) escape latency of Morris water maze test (n = 6). Data are presented as mean ± SD. Statistical significance was tested by one-way ANOVA with Tukey’s multiple comparisons test.


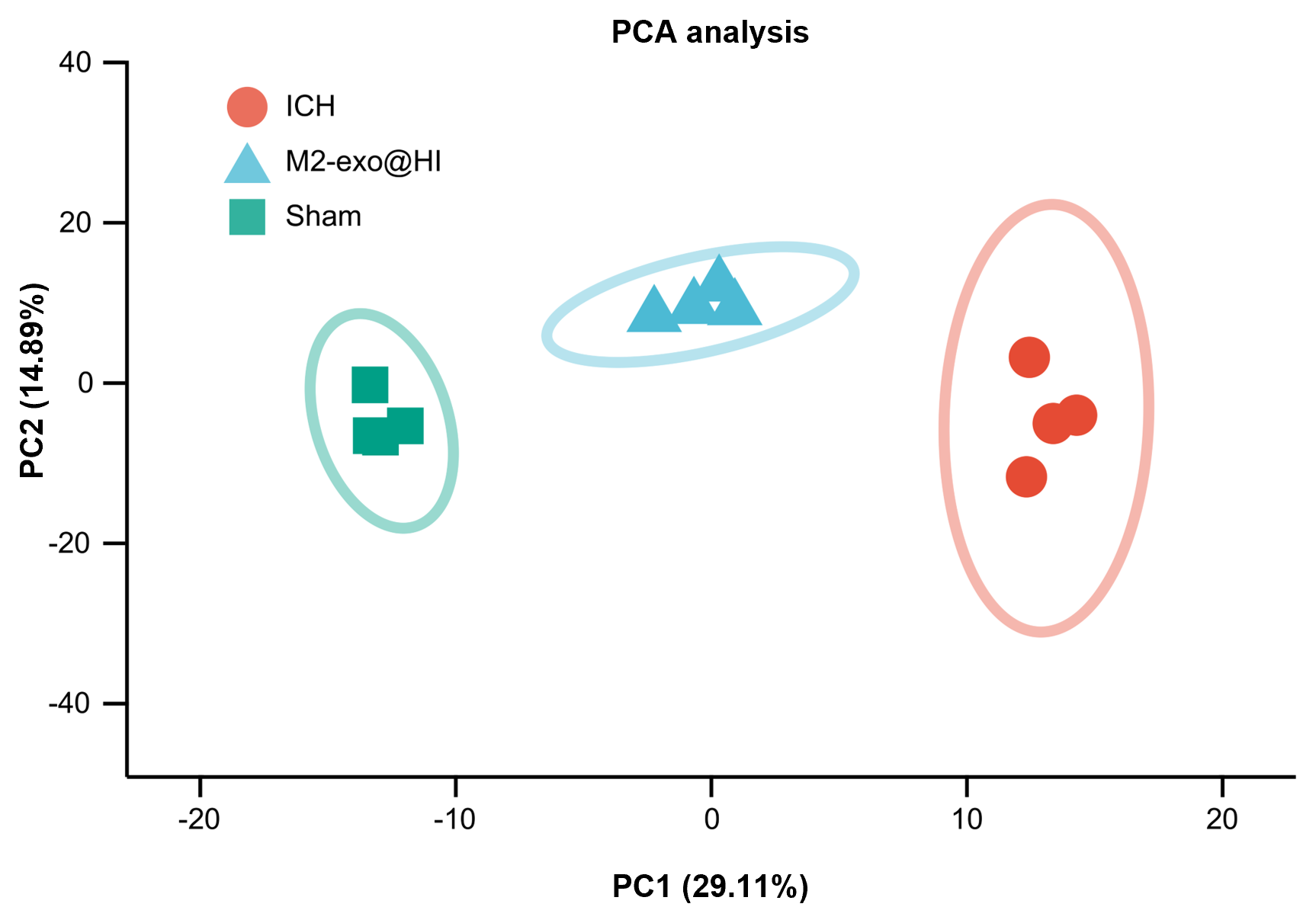


**Fig. S29.** Principal component analysis (PCA) revealed distinct clustering patterns among sham, ICH, and M2-exo@HI groups.


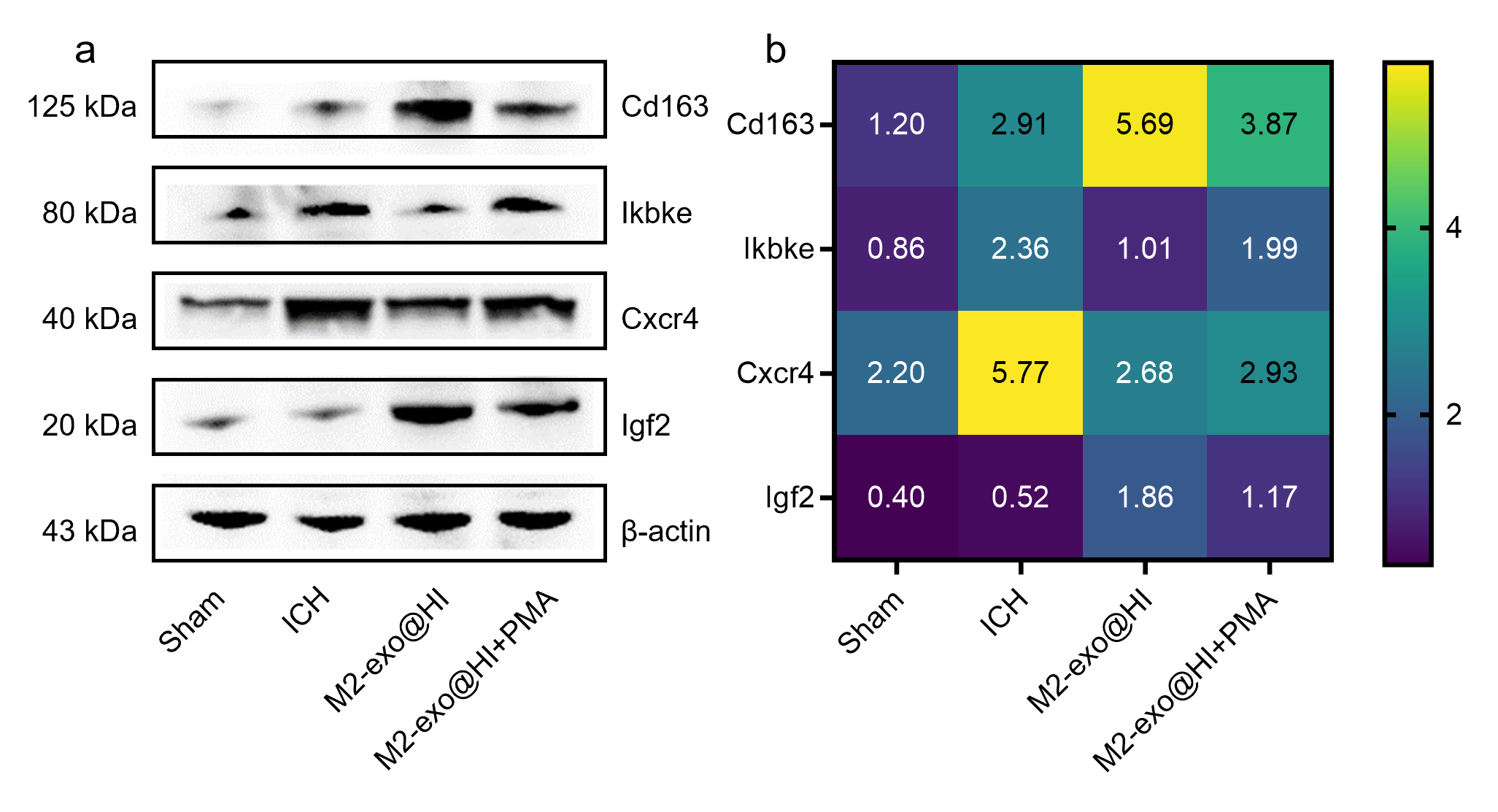


**Fig. S30.** (a) Representative WB iamges of Cd163, Ikbke, Cxcr4 and Igf2 in peri‑hematomal brain tissues from Sham, ICH, M2‑exo@HI and M2‑exo@HI+PMA groups. (b) Heat map summarizing the relative protein expression levels normalized to β‑actin (n = 3).


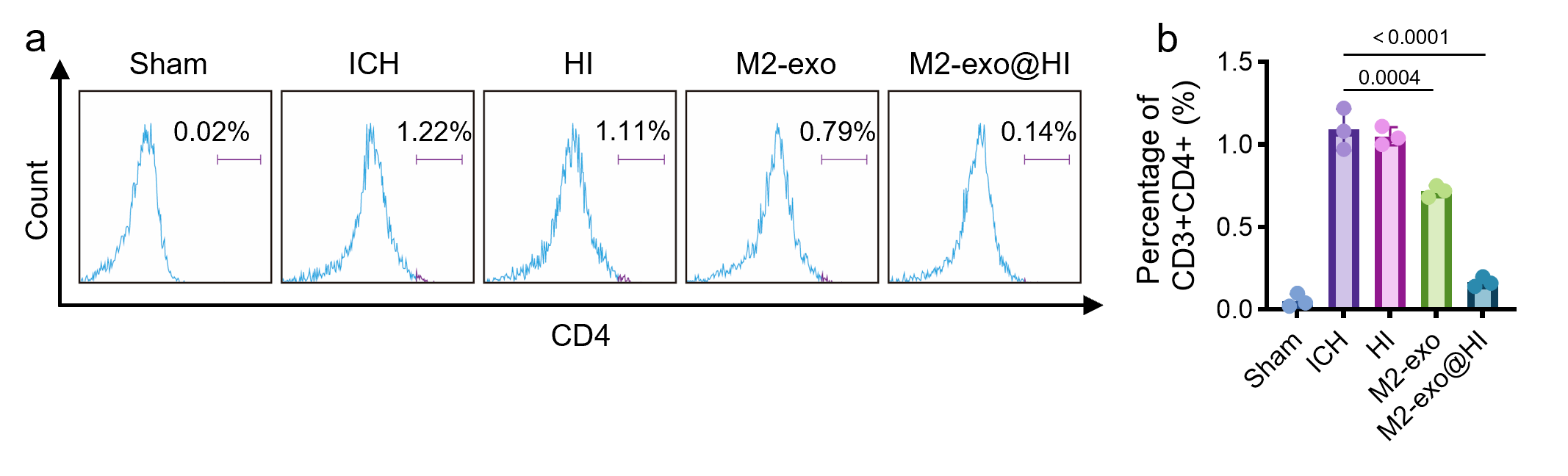


**Fig. S31.** (a) Representative flow cytometry plot of the CD4^+^ T cell in different groups. (b) Quantitative measurements the percentages of CD4^+^ T cells in different groups (n = 3). Data are presented as mean ± SD. Statistical significance was tested by one-way ANOVA with Tukey’s multiple comparisons test.


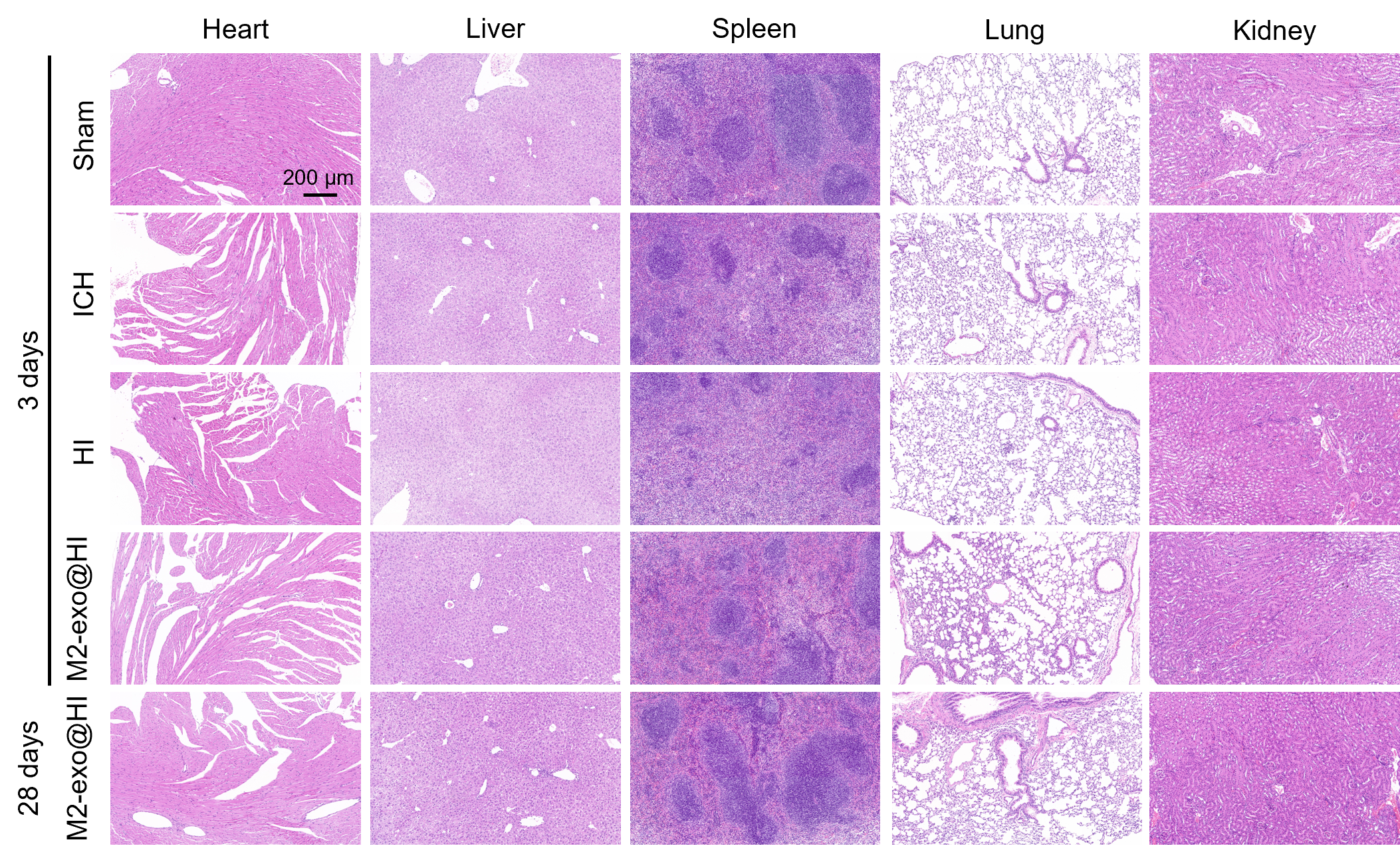


**Fig. S32.** HE staining of heart, liver, spleen, lung and kidney harvested from mice in different groups.


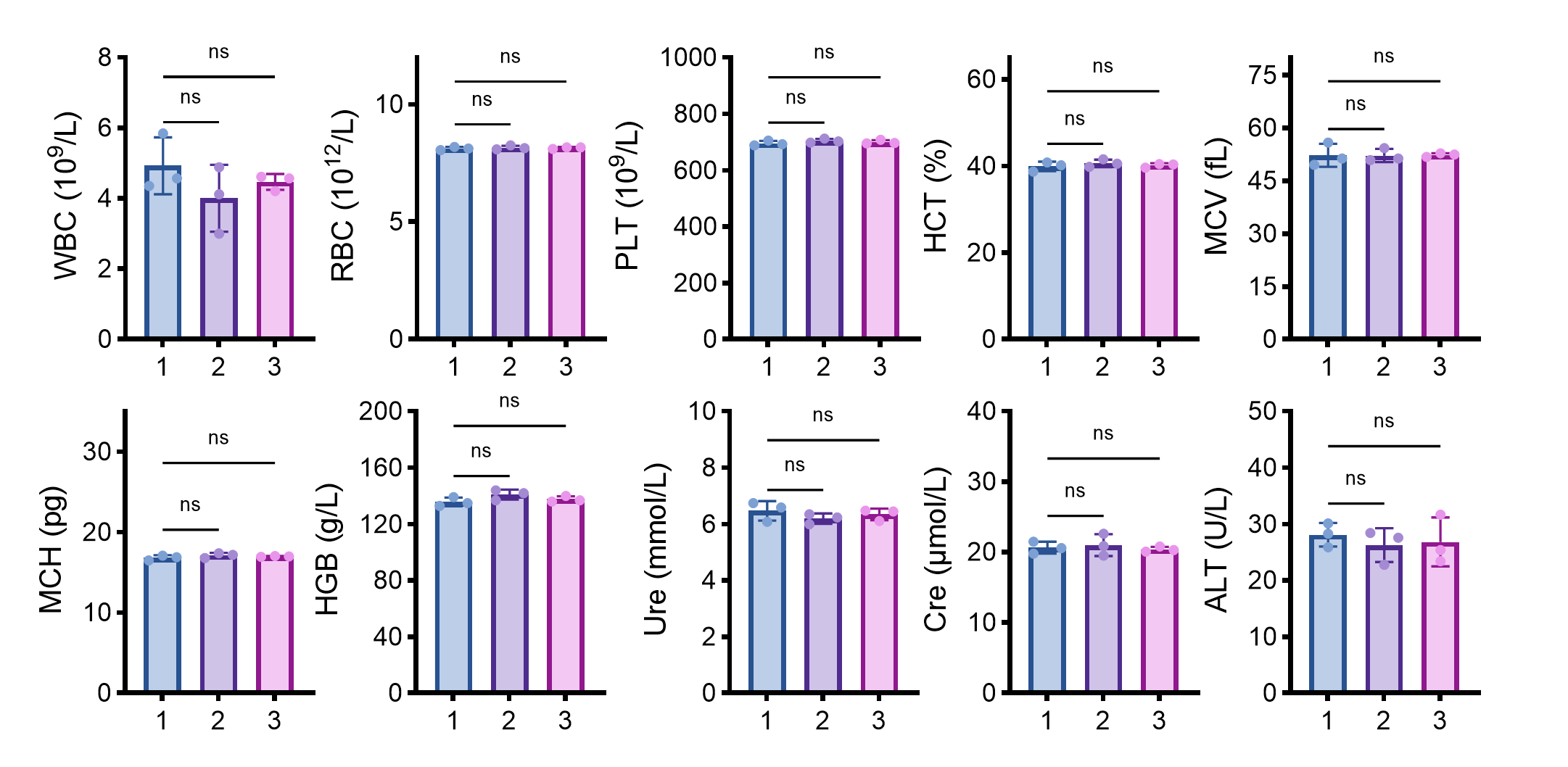


**Fig. S33.** Analysis of blood count and liver and kidney function of mice treated with different groups. 1, 2, and 3 represent PBS for 3 days, M2-exo@HI for 3 days, and M2-exo@HI for 28 days, respectively (n = 3). Data are presented as mean ± SD. Statistical significance was tested by one-way ANOVA with Tukey’s multiple comparisons test.


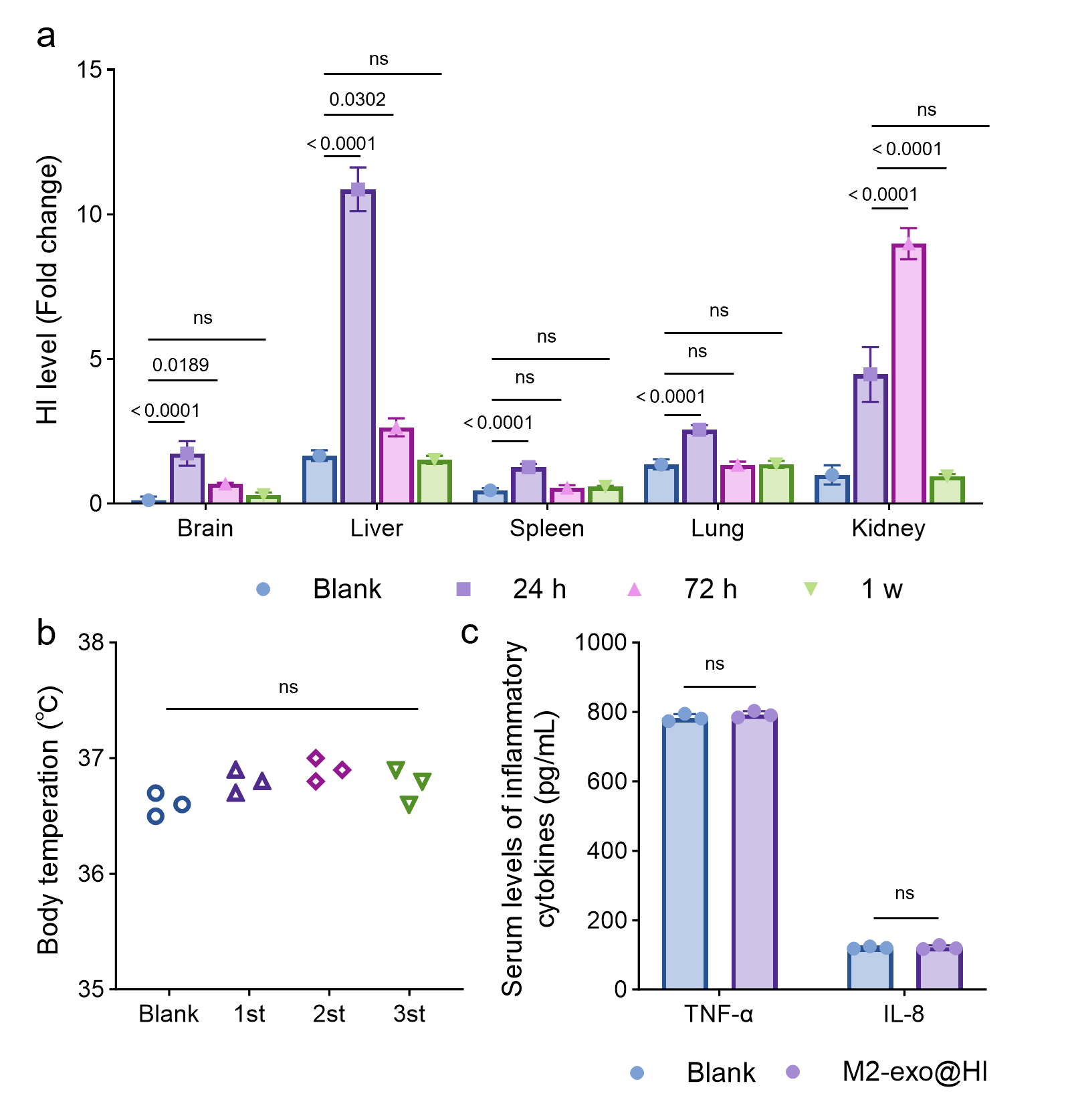


**Fig. S34.** The long-term safety and persistence of plasmid expression in mice. (a) qPCR analysis of HI plasmid clearance in mouse tissues at different time points post-injection (n = 4). (b) Body temperature of mice at 30 min after the first, second, and third injections of M2-exo@HI (n = 3). (c) TNF-αand IL-8 levels in serum at 15 min after the third dose with M2-exo@HI (n = 3). Data are presented as mean ± SD. Statistical significance was tested by one-way ANOVA with Tukey’s multiple comparisons test or unpaired Student’s *t*-test.


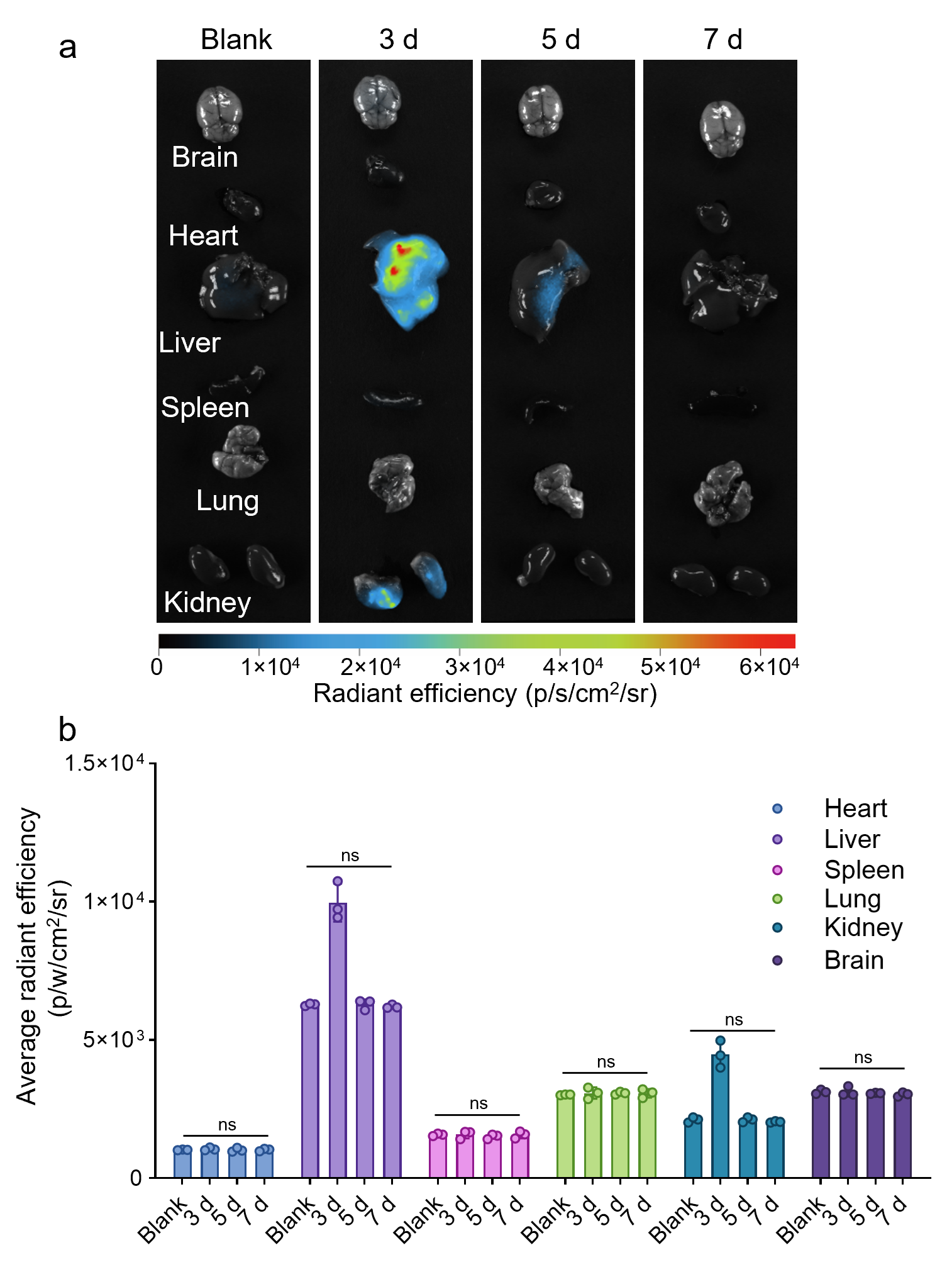


**Fig. S35.** The long-term clearance of M2-exo@ICG in different tissues. (a) Ex vivo fluorescence imaging of M2-exo@ICG in mouse tissues at different time points post-injection. (b) The average radiation efficiency of M2-exo@ICG in mouse tissues based on fluorescence intensity (n = 3). Data are presented as mean ± SD. Statistical significance was tested by one-way ANOVA with Tukey’s multiple comparisons test.
